# Experimental and ML-assisted optimization of injection timing and EGR in a diesel engine fueled with palmyra biodiesel

**DOI:** 10.1039/d5ra05659d

**Published:** 2026-01-30

**Authors:** Jayashri N. Nair, T. Srinivasa Rao, M. B. S. Sreekara Reddy, V. Dhana Raju, Harish Venu, Ahmed Adnan Hadi, Aseel Smerat, T. M. Yunus Khan, Abdul Saddique Shaik, Md. Amir Khan

**Affiliations:** a Department of Mechanical Engineering, VNR Vignana Jyothi Institute of Engineering and Technology Hyderabad Telangana 500090 India jayashri@vnrvjiet.in; b Department of Mechanical Engineering, Vasireddy Venkatadri Institute of Technology Namburu 522508 AP India sr.tanneeru@gmail.com; c Department of Mechanical Engineering, Lakireddy Bali Reddy College of Engineering Mylavaram 521230 AP India khanresearch12345@gmail.com dhanaraju.v@lbrce.ac.in; d Department of Mechanical Engineering (Thermofluids Division), Universiti Teknologi Mara (UiTM) Malaysia 40450 harishvenu@uitm.edu.my harishvenuresearch@gmail.com; e Artificial Intelligence Sciences Department, College of Sciences, Al-Mustaqbal University Babil 51001 Iraq ahmed.adnan@uomus.edu.iq; f Hourani Center for Applied Scientific Research, Al-Ahliyya Amman University Amman 19328 Jordan smerat.2020@gmail.com; g Central Labs, King Khalid University P.O. Box 960 AlQura'a Abha Saudi Arabia yunus.tatagar@gmail.com; h Department of Mechanical Engineering, College of Engineering, King Khalid University Abha 61421 Saudi Arabia ashaik@kku.edu.sa; i Galgotias College of Engineering Knowledge Park 11 Greater Noida 201310 India khanresearch12345@gmail.com; j Al-Ayen Scientific Research Center, Al-Ayen Iraqi University, AUIQ P.O. Box: 64004 An Nasiriyah Thi Qar Iraq

## Abstract

Growing environmental concerns and stricter emission regulations have intensified the need for cleaner combustion and sustainable energy solutions. In this pursuit, Palmyra Methyl Ester (POME) stands out as a promising biodiesel, offering renewable origin and desirable fuel characteristics for cleaner, more sustainable engine applications. This study presents an integrated experimental and computational investigation into the performance, combustion, and emission characteristics of a diesel engine operating on POME blends, with a focus on optimizing injection timing and exhaust gas recirculation (EGR). Using a desirability-based multi-objective optimization framework, engine tests were conducted under varied conditions, guided by Response Surface Methodology (RSM). The predictive capabilities of RSM were benchmarked against advanced machine learning models like Extreme Gradient Boosting (XGBoost) and Random Forest. The optimal setting, found as POME20 with 23°bTDC injection timing and EGR, further improved BTE and significantly lowered NO_*x*_ emissions. Among the predictive models, XGBoost outperformed RSM and Random Forest, yielding the highest test *R*^2^ and lowest MSE and MAPE, demonstrating superior accuracy in predicting engine responses. These results highlight the synergistic potential of renewable fuel utilization and data-driven modeling in optimizing diesel engine operation. The findings provide a viable pathway toward cleaner, high-efficiency combustion systems, contributing to the broader goals of sustainable transportation and global energy transition.

## Abstract 2

Growing environmental concerns and stricter emission regulations have intensified the need for cleaner combustion and sustainable energy solutions.

## Graphical abstract

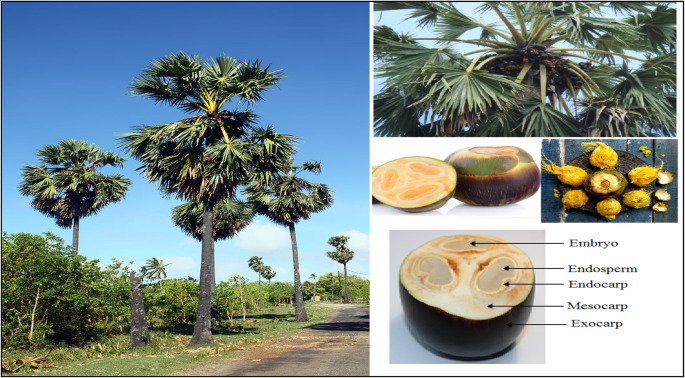

## 1 Introduction

The rising population through industrialization, urbanization and economic growth will lead to the increasing demand of energy in the world. Saravanan *et al.*^1^ investigated the effect of EGR on the combustion dynamics in the presence of enhanced timing of the fuel injection and they found out that the delay period of all the fuels was proportional to the brake mean effective pressure (BMEP) and this effect was due to the increased vaporization of the fuels with the increased levels of brake mean effective pressure, therefore reducing chemical delay. In addition, with AIT the delay time showed about 15 percent extension compared to normal injection time. The exhaust gas recirculation that was introduced in the AIT further increased the mixing period by about 6 percent, mainly because of the reduced oxygen concentration that was caused by the EGR. At the same time, peak pressure showed a positive relationship with BMEP increasing, because the more fuel burned, the more the energy was emitted and, as a result, the higher the cylinder peak pressure became. Shi *et al.*^2^ indicated that an almost isobaric curve of cylinder pressure appeared at increased rates of EGR leading to a passage into low temperature combustion (LTC) mode with an easily identifiable second-stage combustion delay manifested in the HRR curve. EGR was found to be a good technique to reduce NO_*x*_ emission from diesel engines with EGR rates of 10% and 61% in this study. It was noted that the period of combustion delay extended when the EGR rate augmented and extends combustion phase and delayed CA50. This phenomenon was attributed to reduction of intake oxygen concentration owing to the entry of hot exhaust gas of large specific heat capacity, which collectively contributed to lower cylinder temperatures conducive to reduced NO_*x*_ emissions.

Ravindra *et al.*^3^ reported that biodiesels that were formed using various feed stocks generally have the high kinematic viscosity, which affects the atomization of fuel droplets while injected to combustion chamber. The general issues that cause delays in ignition due to higher oxygen levels due to peak combustion temperatures and also known for lower calorific values. Water-in-diesel emulsion fuel was used to overcome the in-cylinder temperatures and improve the performance. Loganathan *et al.*^4^ examined the utilization of a blend of diesel and cashew nut, to optimize the performance and minimize emissions. Additionally, EGR is employed at a rate of 10% to further mitigate NO_*x*_ emissions. Optimization of engine performance is achieved through the identification of an optimal combination of factors, namely 10% exhaust gas recirculation, 6 liters per minute (lpm) hydrogen flow rate, and 20% biofuel blend. Ahmad *et al.*^5^ performed the comparative analysis of microalgae and palm oil as the source of biodiesel feedstocks; they paid attention to the sustainability and environmental impact. It concludes that microalgae would be more sustainable than palm biofuel. The inefficiencies and sustainability issues that are linked to food crops as the source of bio-diesel production have triggered the interest in microalgae as a source of renewable energy. It is the case that microalgae have been recognized as one of the top candidates in the production of biodiesel of the third generation due to the many benefits as a sustainable feedstock.

The optimal blend of SME20 was found by al-Dawody and Bhatti^6^ to be the appropriate blend to implement a diesel engine without any negative consequences. The 19 compression ratios has better engine characteristics. Das *et al.*^7^ had concentrated on the use of castor biodiesel and at the highest load B10 (10% biodiesel mixture) had a similar BTE to that of diesel and the recorded value was 34.8%. Nonetheless, BTE declined as the concentrations of the biodiesel increased and B100 recorded the lowest as a result of its LCV. Manieniyan *et al.*^8^ aimed at to investigate the emissions, vibration characteristics and the effects of EGR on performance at different levels. The application of 10 percent EGR ratio showed a considerable reduction of NO_*x*_ by 21.06 percent at peak load. Agarwal *et al.*^9^ have indicated that the most commonly used method was the EGR that sought to reduce the emission of NO_*x*_ by suppressing the charge presented in the combustion chamber. Experimental tests were done to determine engine performance and emissions under different EGR rates. There was slight increase in thermal efficiency and BSFC reduction at lower loads when using EGR and reduction in exhaust gas temperatures. Harish Venu *et al.*^10^ have examined the issues of increased exhaust emission related to the use of Exhaust Gas Recirculation (EGR). To overcome the challenges, the study proposes the use of a blend comprising palm biodiesel (PB), supplemented with 25 ppm TiO_2_ nanoparticles (PBN).

Murat and Aydin^11^ used the DEE with biodiesel at varying concentrations (2.5%, 5%, 7.5%, and 10% by volume) to create ternary fuel blends. Results inferred that BTE was lowered by 17.39%. However, the ternary blends demonstrated reductions in hydrocarbon, smoke, and nitrogen oxides emissions by up to 12.89%, 4.12%, and 8.84%, respectively over the diesel fuel. Rajan *et al.*^12^ investigated the effects of different injection timings on the diverse attributes of 20% biodiesel blend and they found that YOBD20 with a delayed IT of 21°bTDC and a penalty of 0.896% BTE, NO*x* emission was decreased by 32%, while the other emissions rose in comparison to diesel at the original 23°bTDC.

Subbarayan and Senthil Kumar^13^ studied the hot EGR proved effective in reducing NO_*x*_ emissions, and they found reductions of 14.23% for B100 at maximum load. Hot EGR also showed lower specific fuel consumption than cold EGR, with B25 having the smallest increase in fuel consumption across loads. Surya Kanth *et al.*^14^ examined hydrogen-enriched honge biodiesel blends in compression ignition engines. The HB20 blend enriched BTE by 2.2% and decreased fuel consumption, along with substantial decreases in CO (21%) and HC (24%) emissions due to cleaner combustion. Ashok *et al.*^15^ demonstrated better brake thermal efficiency, with improvements up to 12% for LPO 100, and reduced brake-specific fuel consumption by 1.5–9.0%. These results point to the potential of LPO as a diesel alternative, especially in such locations as India. Hyun Kyu Suh and Chang^16^ put more emphasis on the unique fuel characteristics of biodiesel such as energy content, excess oxygen that improves the combustion phenomenon. Verma *et al.*^17^ found out that diesel engine has demonstrated greater BTE and reduced emissions at higher compression ratio using 10 percent EGR. As demonstrated by Veerabadran *et al.*,^18^ addition of carbon nanotubes (CNTs) to biodiesel enhanced the efficiency and quality of combustion and emissions by enhancing the ignition characteristics. Ahamad Shaik *et al.*^19^ applied the 22 : 1 CR enhanced BTE of the MSME20 by 7.4%. The authors found that tamarind seed methyl ester (TSME) at delayed injection timing reduced CO, HC, and SO and increased NO_*x*_ (Ahamad Shaik *et al.*^19^). Kishore *et al.*^20^ used Taguchi method to optimize the intake parameters of TSME20, and the timing at which it is injected was the most effective parameter to influence the performance. Rao and Prasad.^21^ studied the influence of CR and EGR on the various attributes of diesel engine when POME20 blend was employed at various load states. The findings indicate that, there is a growth in the amount of BTE by 6.91 per cent and a decline in the amount of BSFC by 10.2. Ashish Dubey *et al.*^22^ demonstrated that EGR can be used to reduce NO_*x*_ emissions in soybean oil blended biodiesel, with little effects on efficiency at part load. It was reported by Sun *et al.*^23^ that EGR minimized NOx in dimethyl ether–biodiesel mixtures, but at the cost of more extensive combustion time. Shameer and Ramesh^24^ were able to conclude that the further development of injection time at high pressure promoted combustion. According to Shaeef *et al.*,^25^ dairy scum biodiesel that was injected at 26°bTDC had lowest levels of HC and CO emissions. Jayabal *et al.*^26^ investigated the influence of hydrogen/sapota seed biodiesel in dual-fuel mode, where hydrogen was used as a supplementary fuel. They found notable increases in BTE and considerable reductions in CO, CO_2_, HC, and smoke emissions but with increases in NO_*x*_. Kesharvani *et al.*^27^ Showed that enriching algal biodiesel/diesel blends with hydrogen at volumetric fractions of 5–20% improved combustion by raising peak cylinder pressure and HRR. Emissions of CO, HC dropped significantly with increasing hydrogen share, though NO_*x*_ increased. Noorollahi *et al.*^28^ examined the use of biodiesel/bioethanol blends with nanoparticle additives. He used graphene nanopowder and displayed an increase in power and torque, with decrease in CO, UHC (unburnt hydrocarbons), and NO_*x*_ emissions compared to conventional diesel. Gad *et al.*^29^ found that the hybrid nanoparticle additives also reflect promising results. One such work examined different configurations of TiO_2_, Al_2_O_3_, and hybrid TiO_2_ + Al_2_O_3_ nanoparticles blended with WCO biodiesel (B20); the hybrid nano additives showed up to 12.5% improvements in BTE and significant decreases in CO, HC, and BSFC at full load. A recent experimental study by Patil *et al.*^46^ on palmyra oil biodiesel blends in a CI engine similarly concluded that POME20 is “the most promising blend,” capable of enhancing engine performance while reducing harmful exhaust emissions relative to diesel and higher POME fractions. Similarly, preliminary palmyra biodiesel studies on POME10, POME20, and POME30 consistently show that POME20 offers the best compromise between brake thermal efficiency and emission reduction in CI engines, and is therefore recommended as the optimum blend for practical application reported by Rao *et al.*^47^ and Nair *et al.*^48^

Palmyra seeds, harvested from the palmyra tree, are widely available across many regions of India and contain approximately 40–45% oil. Nevertheless, a deficiency of technical literature on using palmyra seed oil in the biodiesel industry and a deficiency of exploration of the usage of the same in the CI engines is experienced. The research paper discusses Palmyra Oil Methyl Ester (POME20) as an alternative to the biodiesel in terms of its combustion characteristics and emission control. This will be achieved by adding EGR 5% and 10% in order to reduce the emission of NO_*x*_ without compromising engine performance in the research. Compared to other literature, the study offers a solution to the optimization of injection timings and rate of EGR, which is specific to palmyra biodiesel, and thus it fills a research gap in the literature on sustainable fuels.

## 2 Material and methods

**Fig. 1:**
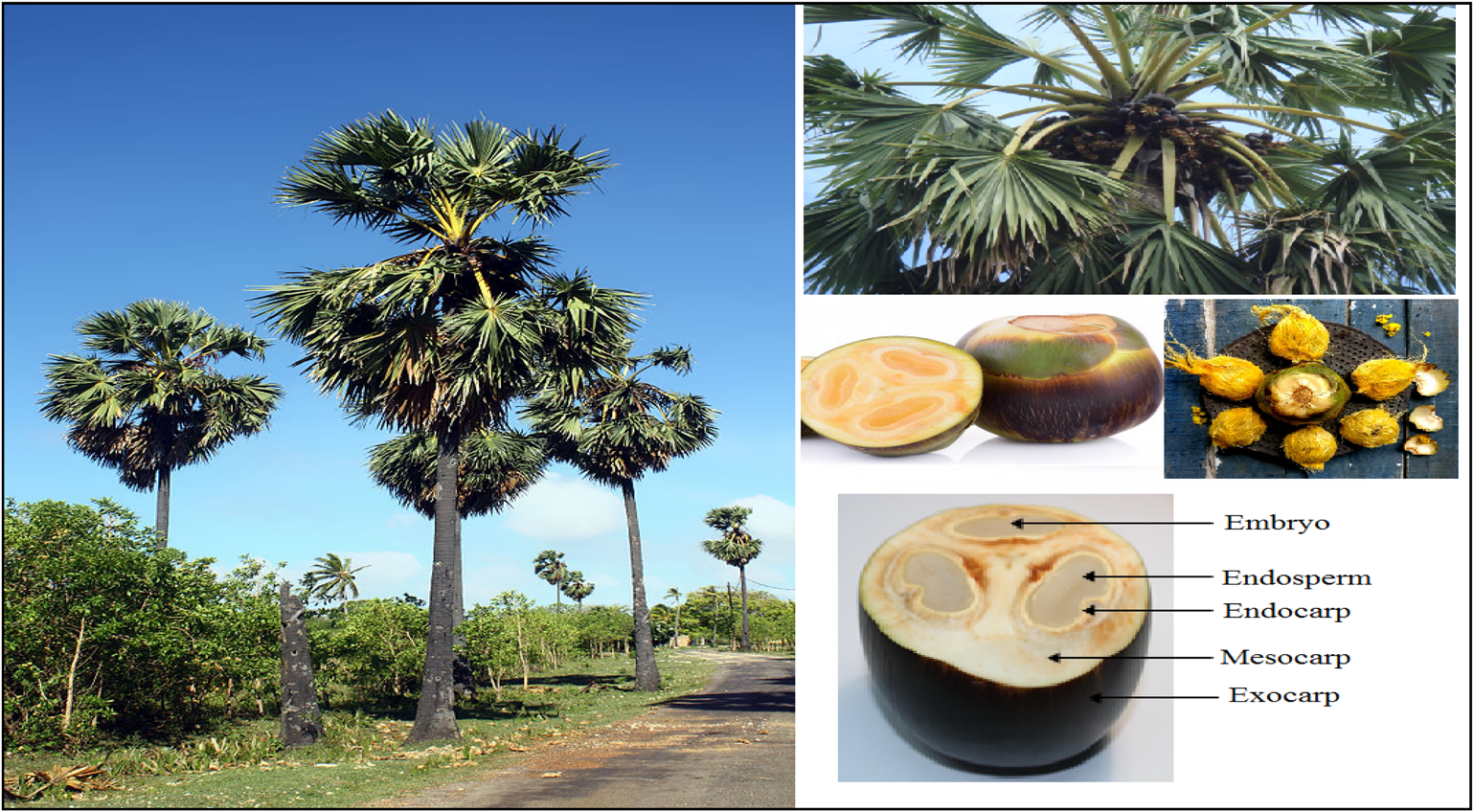
Palmyra trees, fruits and seeds.^21^

Palm palmyra or *Borassus flabellifer*, is a plant named after Greek origin, *Borasus*. This is a widely spread tree that is mostly found in the Indian subcontinent. The world manufacture of palmyra is about 140 million every year with India contributing about 7 million to this manufacture. Palmyra palms are very prolific in the coastal areas of India, the northern part of Sri Lanka and the mainland Sabkha area of Southeast Asia, and flourish in low sandy areas and in open savannah. They are also available in hilly areas with elevation of between 500 and 800 meters. The trunk of the tree can be 1 meter in diameter and reach heights of 25 to 30 meters in height. The trees have 30 to 40 large fan-shaped foliages, and the diameter of the leaves is 1 to 1.5 meters. The flowers produced by the female plants are spherical fruits that are 15 to 20 centimeters in diameter and weigh about 1.5 kilograms on average. An adult Palm tree yields 200 to 300 fruits a year. The fruits are originally green but turn to a rich purple or almost black when they are in the process of ripening. Each fruit houses one to three seeds, occasionally four, encased in a tough outer shell. The seeds of the palmyra palm are typically firm, elliptical in shape, measuring about 5 to 7 centimeters in diameter, and ranging in color from dark to light brown. When young, the seed's endosperm is soft, gelatinous, and sweet, but as the seed matures, it hardens, resembling ivory with a hollow core. The palmyra palm stands as a remarkable symbol of resilience and abundance, deeply woven into the cultural and ecological fabric of the regions it inhabits. palmyra trees, fruits and seeds are displayed in Fig. 1.

Palmyra palm is extremely helpful and is a significant tree in India. Each component is beneficial in some manner. Its leaves may be used to make mats, baskets, fans, hats, and umbrellas, as well as for writing. Its black trunk is robust and sturdy, making it ideal for building and bridge construction. Palmyra palms may be used to make a variety of goods. Palmyra palm stalks are used to construct fences. It is well-known in India for its 800 applications. Sap may be obtained by tapping the inflorescences and then consumed. Additionally, sap can be converted to sugar. Toddy is a beverage made from sap that has been fermented. The seedlings of the palmyra palm are a rich source of starch and may be eaten either raw or cooked. The fruit's endosperm is mushy and gelatinous when freshly eaten. Young palmyra seeds are referred to as ice apples or palmyra fruit seeds, which are most frequently referred to as “Nungu”. The immature seed is eventually hardened and develops a fibrous kernel which aid in oil extraction.

**Fig. 2:**
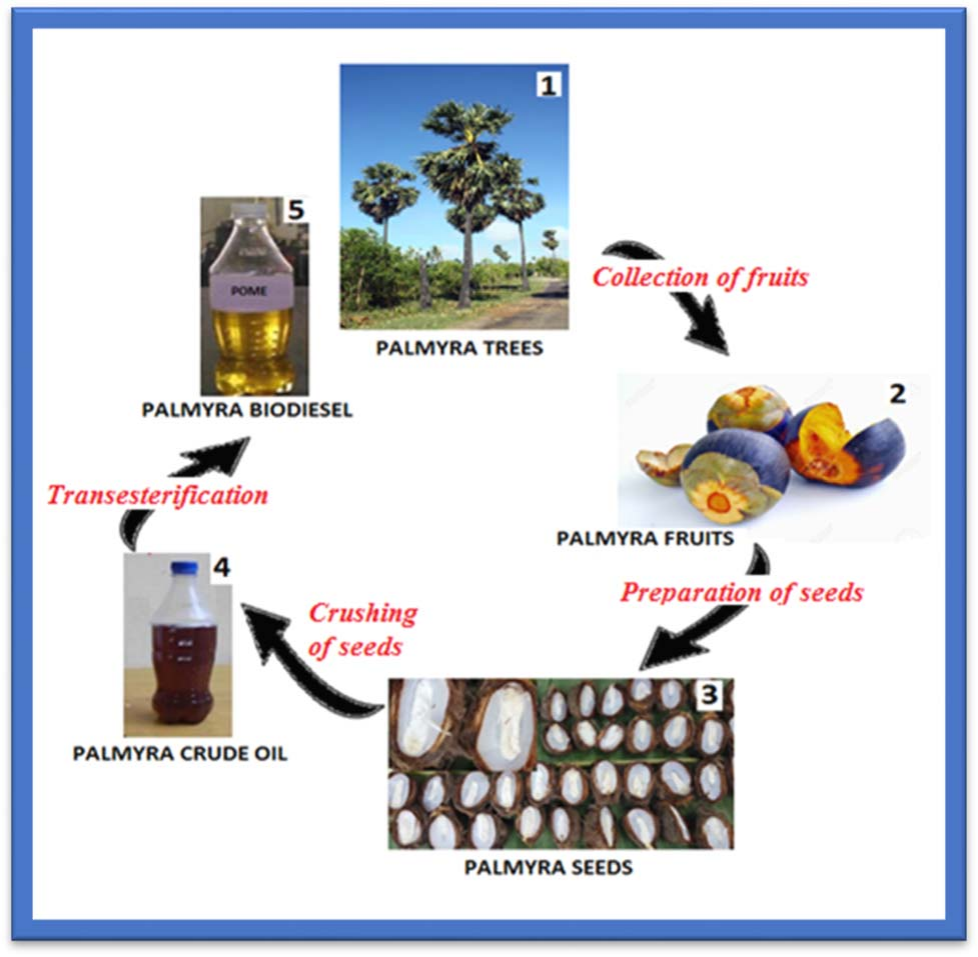
Palmyra biodiesel extraction sequence.^21^

The seeds are washed, sun-dried to reduce moisture, and then crushed to obtain crude oil, which is highly viscous and less prone to evaporation. Through the use of the transesterification method this problem was greatly attenuated. These steps are shown in Fig. 2, for biodiesel production in palmyra.

### 2.1 Transesterification

**Fig. 3:**
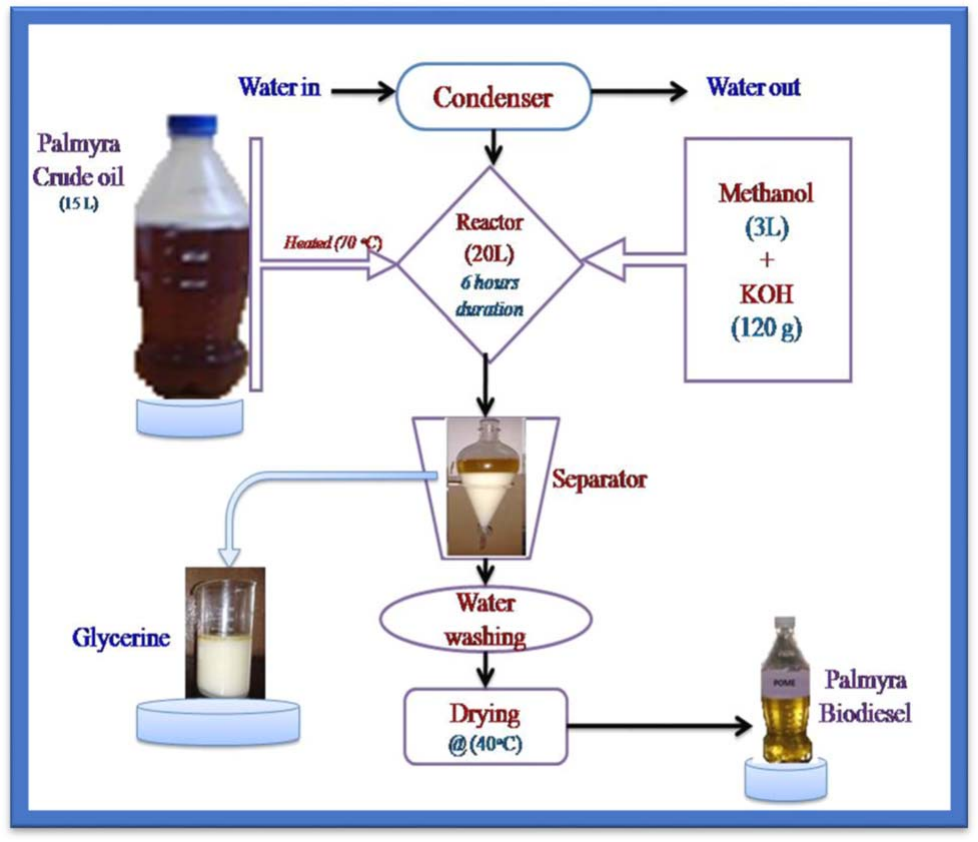
Palmyra biodiesel extraction through the transesterification process.^48^

Transesterification is a chemical process that converts vegetable oils into biodiesel by reducing their viscosity and improving fuel properties. In the presence of a catalyst, triglycerides in the oil break down into glycerol and fatty acid esters through three stages, producing three ester molecules per triglyceride.^30^ Several important factors affect the process with fatty acid content, type and concentration of the catalyst, alcohol and oil molar ratio, reaction temperature, mixing intensity being some of the major ones. This changes vegetable oils to be more appropriate in use as fuel. These are the major drawbacks of unrefined palmyra oil because it is less volatile, with high viscosity, and density. Fig. 3 shows the extraction of palmyra biodiesel using the transesterification process. Crude palmyra oil is highly viscous, low volatile, and does not have high density and therefore, it is necessary to undergo transesterification to enhance the quality of the product. The mixture of palmyra oil (15 liters), methanol (3 liters) and KOH catalyst (120 grams) is stirred during a period of 180 minutes at 70 °C. The mixture then left to rest during 24 h and two distinct layers were formed; glycerin at the bottom and biodiesel at the top. The result of separation is the washing and drying of the biodiesel that produces approximately 90%. Table 1 displays fuel properties.

**Table 1:** Physical and chemical properties of diesel and POME biodiesel blends^48^

Properties	Diesel	POME	POME10	POME20	POME30	POME40
Density (kg m^−3^)	830	872	836	841	845	849
Dynamic viscosity (cSt)	3.4	22.2	5.28	7.1	9.0	10.9
Heating value (kJ kg^−1^)	42 500	37 889	42 039	41 577	41 116	40 655
Flash point (°C)	54	169	66	75	88	98
Fire point (°C)	57	172	69	77	91	102
Cetane number	46	52	47	48	50	51

### 2.2 Fatty acids composition of POME

**Fig. 4:**
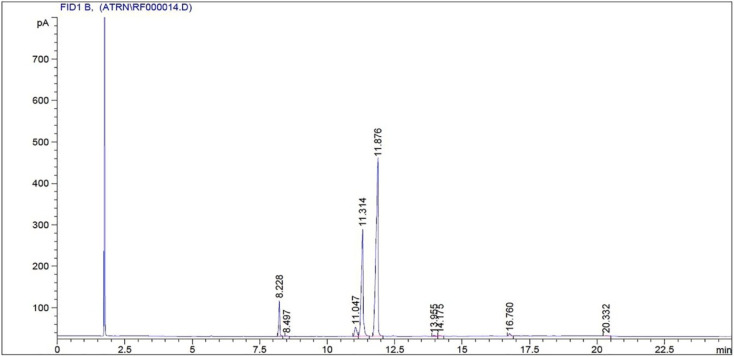
Gas chromatogram of palmyra oil methyl ester.^48^

Using the gas chromatographic method to analyze unblended POME biodiesel, the major fatty acid methyl esters of the product were identified using the retention time (*x*-axis) and the detector response (*y*-axis), with the area of the peak giving a relative compositional value. The GC-MS analysis revealed that POME biodiesel is primarily composed of linoleic acid methyl ester (C18 : 2, 58.4%); oleic acid methyl ester (C18 : 1, 31.0%); palmitic acid methyl ester (C16 : 0, 6.0%); and stearic acid methyl ester (C18 : 0, 3.2%). The preponderance of unsaturated ester (linoleic and oleic acids) features improve the atomization, cold flow properties and combustion efficiency which proved to be less CO and HC emissions. On the other hand, the low levels of saturated esters (palmitic and stearic acids) have a small effect on the quality of ignition but lead to lower oxidation stability. As indicated in Fig. 4, the ratio of the dominance of linoleic acid is the highest and stearic acid the lowest. In general, the high unsaturation of POME biodiesel contributes to the efficient and cleaner burning process, which is in line with the previous results on the properties of biodiesel fuels. These compositional trends and effects on the combustion have been recorded in other studies including Vellaiyan S^31^ who optimized *Spirulina* biodiesel ammonium hydroxide blends with EGR to optimize atomization and NOX effects, and Vellaiyan S^32^ who reported improved oxidation and emission reduction in *Bauhinia malabarica* biodiesel diesel blends with electrostatic precipitators. These findings collectively affirm that the high unsaturation level of POME biodiesel contributes to efficient and cleaner combustion performance in diesel engines.

### 2.3 Varying injection timing

**Fig. 5:**
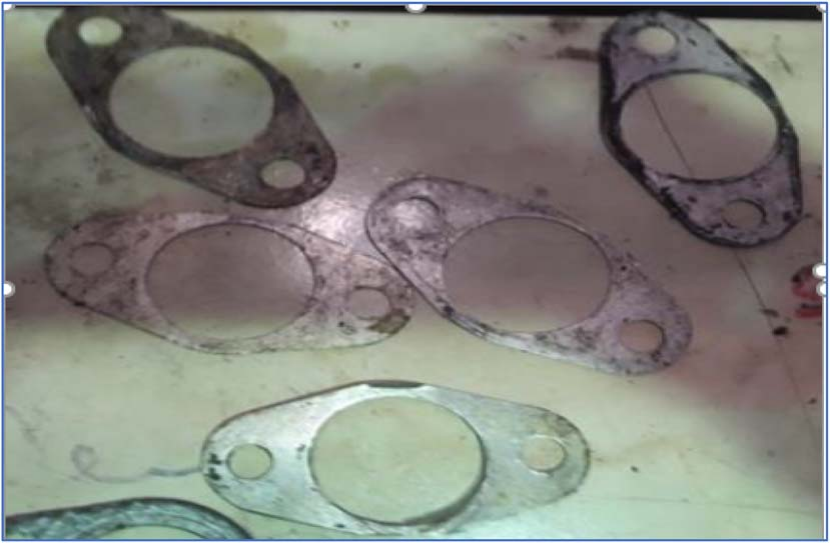
Shims used to vary the injection timing crank angle.

One more operating parameter which influences the engine performance is injection timing. Researchers have studied how injection timing affects engine performance and emissions. Injection timing can be precisely controlled by adjusting the number of shims between the fuel pump and engine. Adding shims delays injection, while removing them decreases the gap, advancing the timing. Similarly, application of shims increases the gap and hence delays the injection timing. Shims that can be inserted to adjust injection timing are illustrated in Fig. 5.

### 2.4 Exhaust gas recirculation (EGR)

Atmospheric nitrogen exists as a stable diatomic molecule, remaining largely unreactive under normal conditions due to low temperatures. The primary causes of NO_*x*_ generation are the combustion chamber's excessive oxygen concentration and high temperatures. NO_*x*_ emissions may be efficiently reduced by adjusting engine operating parameters such as injection time, injection pressure, EGR rates *etc.* Very few reports indicated that biodiesel-fuelled diesel engines reduced NO_*x*_ emissions. Intriguingly, 15% of studies found no difference in NO_*x*_ emissions when using various biodiesel feedstocks in compression ignition engines.

Biodiesel-fueled compression ignition engines emit lower HC, CO, and smoke but produce more NO_*x*_. EGR is efficient approach to lower the cylinder temperature and oxygen content. However, higher EGR rates increase hydrocarbons, carbon monoxide, and smoke opacity. In this study, EGR involves fresh air mixes with exhaust during the suction stroke as shown in Fig. 6.1
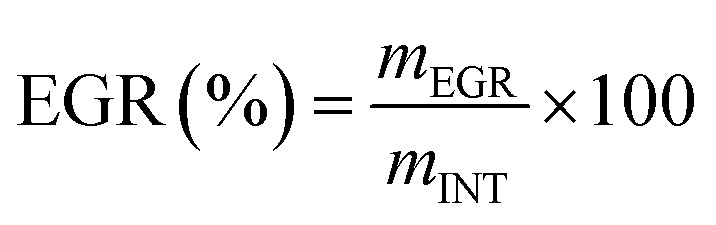


**Fig. 6:**
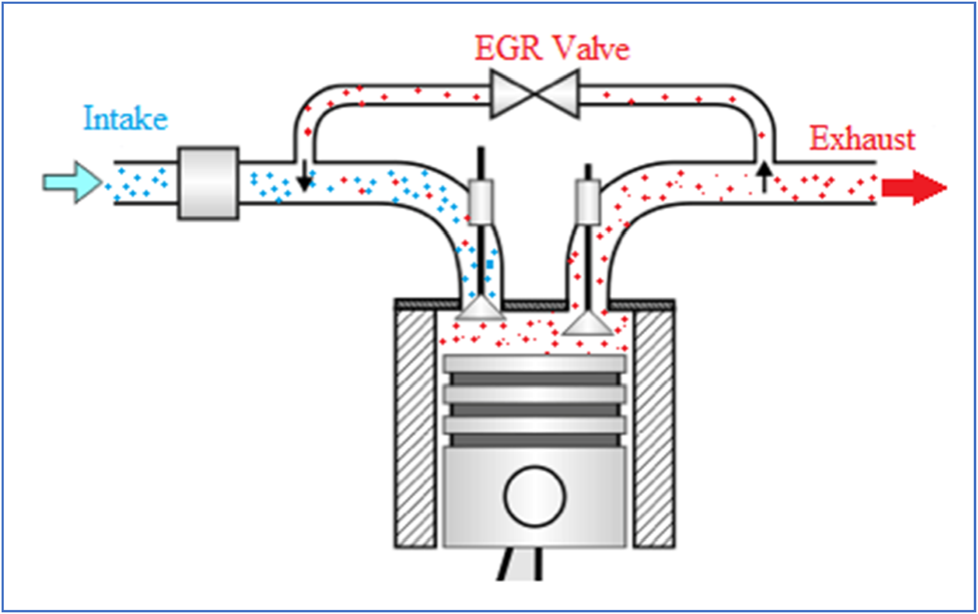
Exhaust gas recirculation system.^36^

In the present study, only 5% and 10% EGR rates were considered because earlier EGR investigations on diesel–biodiesel engines have consistently shown that low EGR levels in the range of 5–10% provide an effective NO_*x*_ reduction with limited increases in HC, CO and smoke, whereas further increases in EGR lead to a rapid deterioration in combustion, thermal efficiency and particulate emissions. In line with these findings, preliminary trials carried out on the POME20 blend in our test engine showed that EGR rates above 10% resulted in unstable combustion and a noticeable drop in brake thermal efficiency, without any proportional additional benefit in NO_*x*_ reduction. Therefore, 5% and 10% were selected as practical and technically meaningful EGR levels that capture the beneficial NO_*x*_–efficiency trade-off region for POME20. These are closely agreement with the results reported by Karthikeyan and Thiyagarajan.^49^

### 2.5 Response surface methodology

Response Surface Methodology (RSM) and desirability-based optimization are widely used techniques in the optimization of complex systems, particularly in engineering, manufacturing, and scientific research. RSM integrates mathematical and statistical methods to model and optimize processes where multiple input variables influence one or more output responses. Usually a second-order polyn. Equation, the method develops an empirical model to forecast the interactions between the factors and the responses.^33^ This is accomplished by means of a series of planned experiments to gather data, thereafter applied to create the response surface. The process starts with the identification of important elements and their ranges *via* first screening. Often used for experimental planning, central composite designs (CCD) or box-behnken designs (BBD) effectively span the design space. The gathered data is fitted to a model, and the fit of the model is assessed by means of analysis of variance (ANOVA). Optimizing methods can be used once a consistent response surface is found.^34^

A technique in RMS called desirability-based optimization turns several response goals into a single desirability function. Every response has a desirability score between 0 (unfit) and 1 (perfect).^35^ A geometric mean aggregates the individual desirability functions to get an overall desirability score. This method helps several contradictory reactions, including optimizing efficiency while lowering cost, to be simultaneously optimized. User-defined goals (*e.g.*, maximize, minimize, or target), weights, and significance levels for every response direct the desirability function. Through iteratively changing the input variables, the procedure finds a set of conditions optimizing the general desirability. In multidisciplinary optimization issues, this approach is very useful since it helps researchers to obtain balanced solutions that fit both theoretical and pragmatic limitations.^36,37^

### 2.6 Machine learning approaches

#### 2.6.1 Random forest

Random Forest Regression is a powerful ensemble learning method that operates by constructing a multitude of decision trees during training and outputting the mean prediction of the individual trees for regression tasks. Inspired by bagging—Bootstrap Aggregating—where every decision tree is trained on a random subset of the input with replacement—this model Random Forest Regression's basic operation starts with the random sampling of data points and features creating several decision trees.^38^ These trees resist trimming since they are grown to their maximum depth, therefore enabling their collection of intricate data linkages. At every split, a subset of characteristics is randomly chosen for every individual tree to guarantee variation among the trees and lower the overfitting risk. This randomization is absolutely essential since it helps to prevent the prejudices connected with single decision trees. Every tree forecasts on its own; the Random Forest model generates its final result by averaging the forecasts made by all the individual trees. Unlike a single decision tree, this aggregating procedure helps lower variance and enhance the generalization capacity of the model.^39,40^

**Fig. 7:**
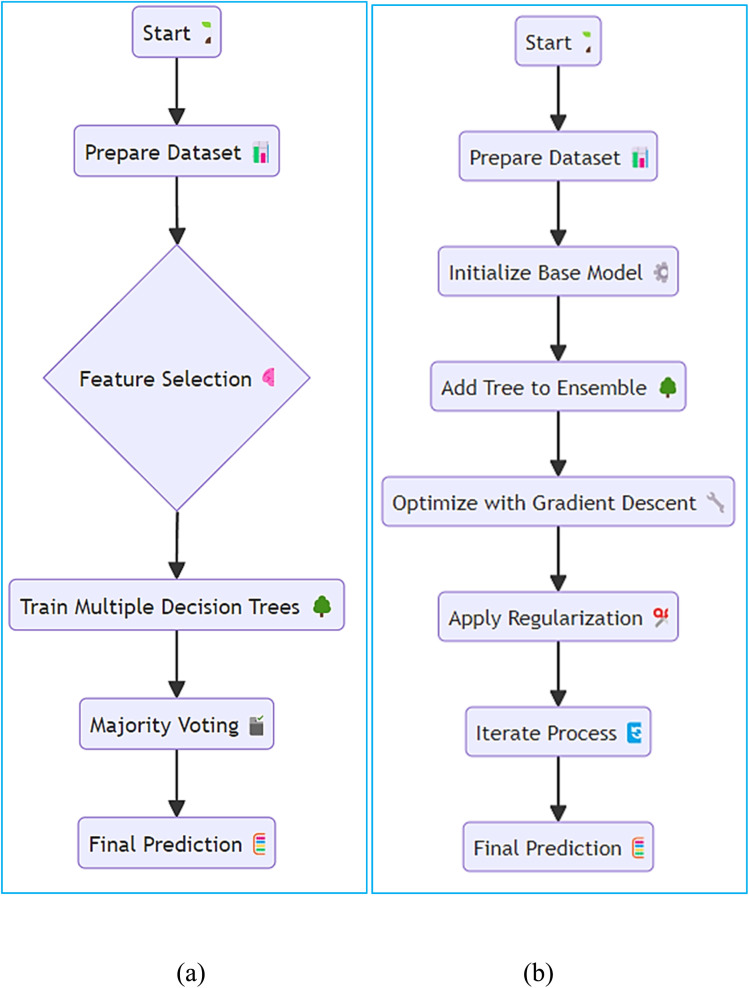
Schematic flow chart of (a) RF (b) XGBoost.

Every tree is built using a criterion such Mean Squared Error (MSE) to assess the split quality. Choosing the appropriate feature and split that lowers the MSE the greatest helps to minimize the error at every decision node. Random Forest Regression performs very well in practice in capturing non-linear correlations between the target variable and the input characteristics.^41^ Random Forest Regression has one major benefit in that it can manage high-dimensional datasets including intricate feature interactions with complex interactions. Average results from several trees help to greatly lower the frequent overfitting risk in decision tree models. Additionally available in the model are feature importance scores, which give insightful analysis of the relative relevance of every input feature in target variable prediction.^42^ Random Forest Regression can be computationally costly, nevertheless, despite its benefits, particularly with large datasets since the model requires the development of several trees and storage of a significant volume of information. Furthermore, lacking the interpretability of a single decision tree, the forecasts can be a restriction in some situations where model transparency is crucial. Still, Random Forest Regression is a useful tool for many real-world projects because of its generalizing ability, resilience, and adaptability. The flow chart of RF is depicted in Fig. 7a.

#### 2.6.2 Extreme gradient boosting

Extreme Gradient Boosting (XGBoost) is a widely used and highly efficient machine learning algorithm that builds an ensemble of decision trees through an iterative process, improving predictions step by step^43^ Based on gradient boosting, a method whereby every new tree is taught to fix errors caused by the past trained trees, faster and more precise than conventional gradient boosting models, XGBoost improves this approach by including regularization techniques and a more effective method of handling the gradients.^44^ Usually set to the mean of the target variable for regression problems, XGBoost starts its operation with an initial prediction. The method then fits a fresh decision tree to the residual errors (gradients) of the model's current predictions at each boosting step. Usually calculated by the mean squared error (MSE) in regression tasks, the tree is built by repeatedly separating the data depending on the characteristic that reduces the error. Focusing on the misclassified data points or residuals, every tree in the sequence fixes the faults of its predecessors.^45^

XGBoost stands out for using regularizing methods—more especially, L1 and L2 regularization—which aid to prevent over fitting and help to control model complexity. These methods reduce too complicated trees and punish big coefficients, therefore enhancing the generalizing capacity of the model. A major benefit in real-world datasets where missing values are widespread is XGBoost's clever strategy to handle missing data: it automatically learns the ideal direction for missing values during tree building. XGBoost is primarily innovative in using second-order derivatives—that is, the Hessian—in the optimization process. XGBoost increases efficiency by considering both first and second derivatives, which produces more accurate updates and faster convergence than conventional gradient boosting employs only first-order derivatives—the gradient. This is accomplished by means of an approximation technique known as “Newton's method,” which improves the estimate of the ideal split points thereby refining the tree-building process. XGBoost also scales the contribution of every individual tree using a technique known as “shrinkage,” sometimes referred to as learning rate. The schematic flow chart of XGBoost is depicted in Fig. 7b.

## 3 Experimental setup

**Fig. 8:**
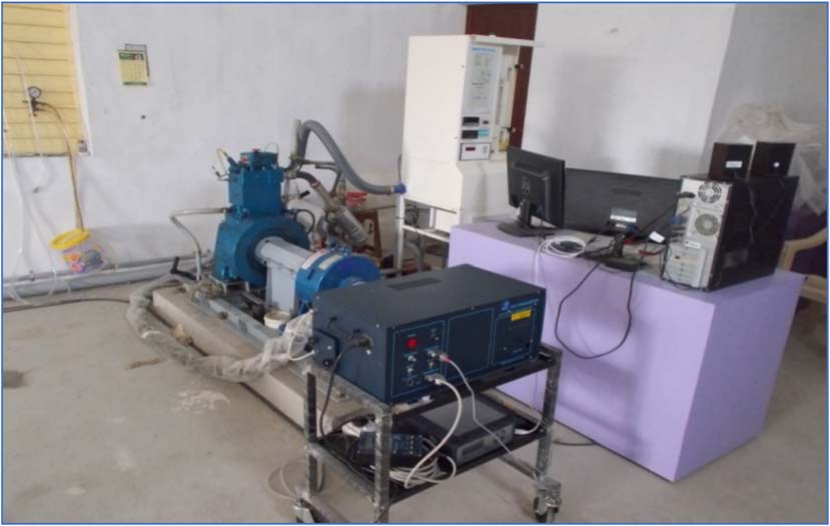
Experimental set-up.^48^

The experimental research was conducted on a single-cylinder CI water-cooled engine, which was loaded at different conditions with a constant 1500 rpm. The engine load was adjusted using an eddy current dynamometer. The experimental design of this study is shown in Fig. 8 and the specification of the diesel engine is given in Table 2. A regulating system allows the engine to stabilize its speed because the load changes. The dynamometer is directly attached to the output shaft of the engine and it provides the necessary load during the test. Moreover, proper instruments were put in place to gauge the various qualities of diesel engine under different loads after proper inspection and calibration.

**Table 2:** Engine specification details

Manufacturer	Kirloskar make
Speed	1500 rpm
Stroke volume	661 cm^3^
Diameter	87.5 mm
Stroke	110 mm
Compression ratio	18 : 1
Diameter of nozzle	0.3 mm
Fuel injection pressure	200 bar

### 3.1 Uncertainty analysis

Any error is determined as any difference between the experimental result and the experimental data, which is usually due to the equipment constraints, calibration, changes in the atmosphere, or the variability in reading. The errors indicate uncertainty and will be presented in Table 3. In order to improve the credibility and repeatability of the experimental findings, uncertainty analysis was applied to all measured parameters. Such analysis is critical in diesel engine testing to guarantee accuracy and credibility because performance and emissions parameters such as BTE, BSFC, and gaseous emissions are determined using several measurements that are subject to sensor errors, calibration drift, and environmental variations. The measurement of systematic and random errors assists in determining the level of confidence around the data and differentiating true trends and noise in the experiment. It is particularly relevant when combining machine learning models, whereby unaccounted measurement errors can bias predictions. Uncertainties reporting and analysis therefore enhance transparency, comparability, and validity of overall performance emission optimization outcomes. Uncertainty analysis serves to determine the measurement accuracy and the precision limits of the engine characteristics. Unknown uncertainties are computed with the following equation.2
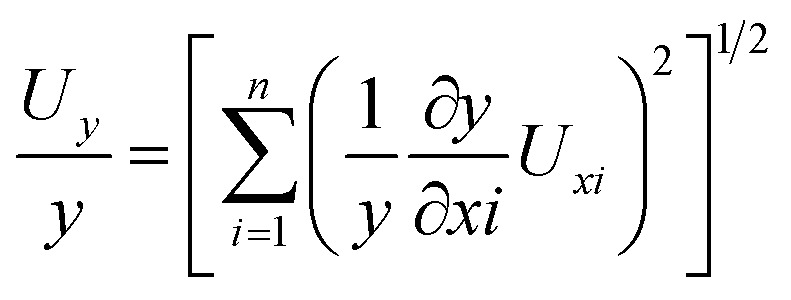


**Table 3:** Range, accuracy and uncertainties of used instruments

Device	Parameter	Range	Accuracy	Uncertainty
Multi gas analyser	HC	0 to 1500 ppm	±10 ppm	±0.2%
	CO	0–10%	±0.03%	±0.2%
	No_*x*_	0–5000 ppm	±10 ppm	±1%
	CO_2_	0–20%	±0.5%	±0.15%
Smoke meter	SO	0–99%	±1%	±1%
Pressure transducer	Pressure	0–100 bar	±1 bar	±0.15%
Temperature indicator	Temperature	0–9000 C	±10 C	±0.2%

The total uncertainty of the test

3 = [(BTE)^2^ + (BSFC)^2^ + (CP)^2^ + (HRR)^2^ + (CO)^2^ + (HC)^2^ + (SO)^2^ + (NO_*x*_)^2^]^1/2^

= [(0.5)^2^ + (0.25)^2^ + (1)^2^ + (1)^2^ + (0.2)^2^ + (0.2)^2^ +(1)^2^ + (1)^2^]^1/2^= ± 2.09%.

## 4 Discussions on results

### 4.1 RSM results

#### 4.1.1 Brake thermal efficiency

**Fig. 9:**
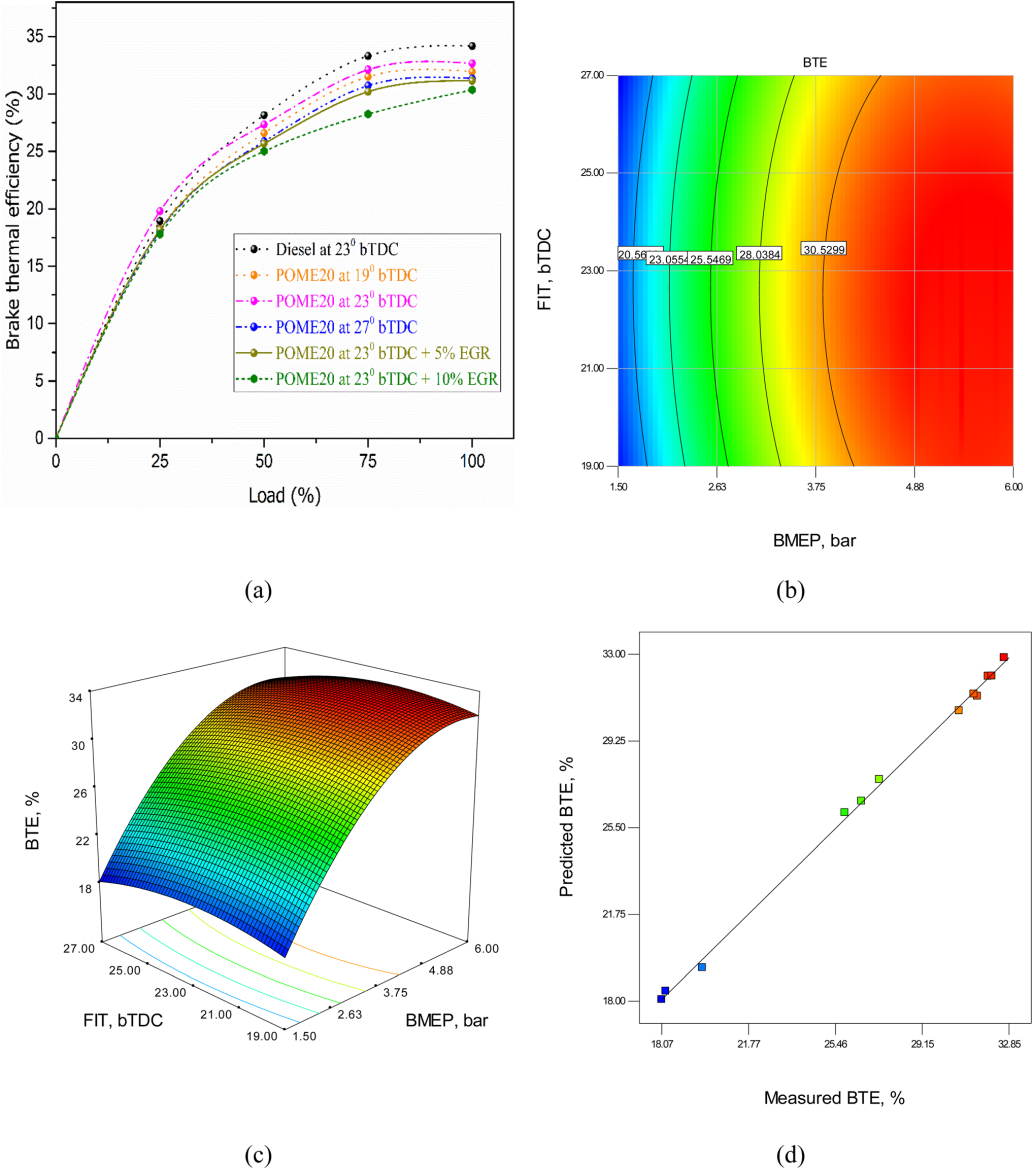
BTE model (a) test results (b) 2D contour plot (c) 3D surface plot (d) predicted *vs.* actual values.

The changes in BTE for the examined fuels with load are shown in Fig. 9. Tuning of the injection timing is the best way of making the process of combustion coincide with the power stroke to achieve maximum energy conversion and better BTE. Earlier injection of fuel results in improved mixing of the fuel with air, which causes a higher level of complete combustion. This tends to amplify low to moderate load BTE but can result in higher load pressure and knocking. A small portion of recirculation of exhaust gases lowers peak combustion temperatures, minimising nitrogen oxide (NO_*x*_) emissions without significant impact on oxygen available to combustion to support increased BTE. Combustion is enhanced at high loads, increasing BTE. Yet, in case of too early injection, the pressure is too high and the energy is lost. Ergonomic rates may also be high at high loads, which can also decrease efficiency due to limited oxygen.

The test results indicate that the brake thermal efficiency (BTE) at peak load for diesel, POME20 at 19°bTDC, 23°bTDC, and 27°bTDC, as well as POME20 at 23°bTDC with 5% and 10% EGR, are 34.35%, 31.92%, 32.06%, 31.36%, 31.15%, and 29.32%, respectively. Similarly, at full load, the BTE values are 34.24%, 31.92%, 32.06%, 31.36%, 31.15%, and 29.32%. The introduction of EGR at 5% and 10% for POME20 at 23°bTDC results in a declining BTE trend with increasing EGR percentage, aligning with findings from Harish *et al.*,^10^ which reported reduced BTE across all load conditions with EGR use.

The RSM equation for BTE is given byBTE = −30.99 + 9.36 × BMEP + 3.39 × FIT − 0.014 × BMEP × FIT − 0.82 × BMEP^2^ − 0.074 × FIT^2^

#### 4.1.2 Brake specific fuel consumption

**Fig. 10:**
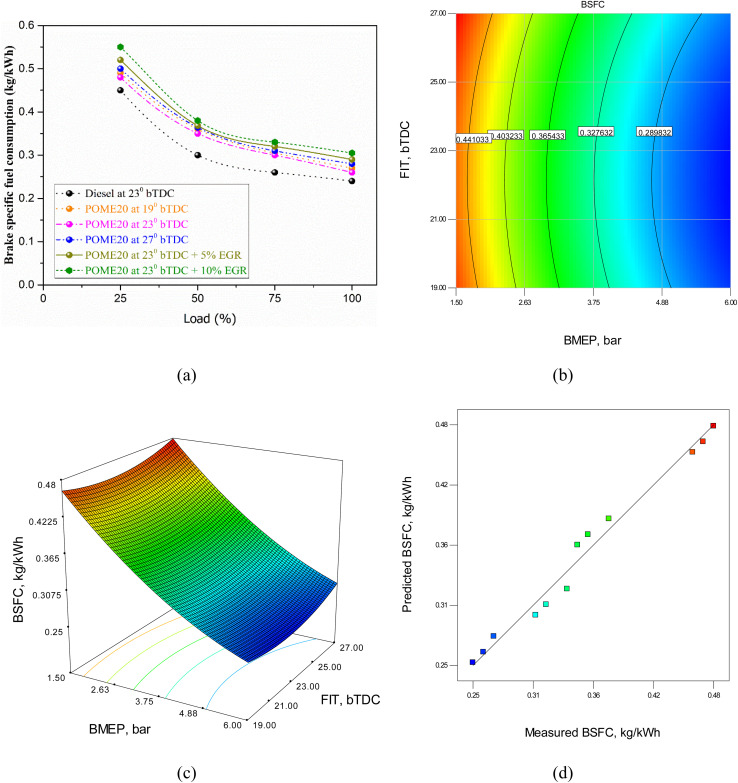
BSFC model (a) model predicted *vs.* actual values (b) 2D contour plot (c) 3D surface plot. (d) Measured BSFC *vs.* predicted BSFC.

The fuel consumption rate per unit of power output for palmyra biodiesel varies with engine load, injection timing, and EGR rates due to changes in combustion efficiency and energy conversion. Fig. 10 illustrates the variation of BSFC with load, primarily influenced by the fuel's energy content and cetane number. At maximum load, diesel exhibits the lowest BSFC at 0.24 kg kW^−1^ h^−1^. For POME20, BSFC is 0.26 kg kW^−1^ h^−1^ at 19°bTDC, decreases to 0.25 kg kW^−1^ h^−1^ at 23°bTDC, but rises to 0.27 kg kW^−1^ h^−1^ at 27°bTDC. Adding EGR at 23°bTDC increases BSFC, reaching 0.29 kg kW^−1^ h^−1^ with 5% EGR and 0.31 kg kW^−1^ h^−1^ with 10% EGR. These results confirm that diesel achieves the highest fuel efficiency, followed closely by POME20 at 23°bTDC without EGR. BSFC decreases with load due to improved combustion efficiency but increases with higher EGR rates due to oxygen reduction, leading to incomplete combustion. Specifically, Vellaiyan *et al.*^31^ found that optimizing water and 1-pentanol concentrations in biodiesel–diesel blends improved combustion and reduced particulate emissions while producing small changes in BSFC and thermal efficiency an outcome that mirrors our observation that oxygenated co-additives improve combustion phasing and reduce smoke but require careful tuning to avoid efficiency loss.

The RSM equation for BSFC is given byBSFC = 1.1 − 0.081 × BMEP − 0.05 × FIT + 0.0048 × BMEP^2^ + 0.001 × FIT^2^

#### 4.1.3 Cylinder pressure

**Fig. 11:**
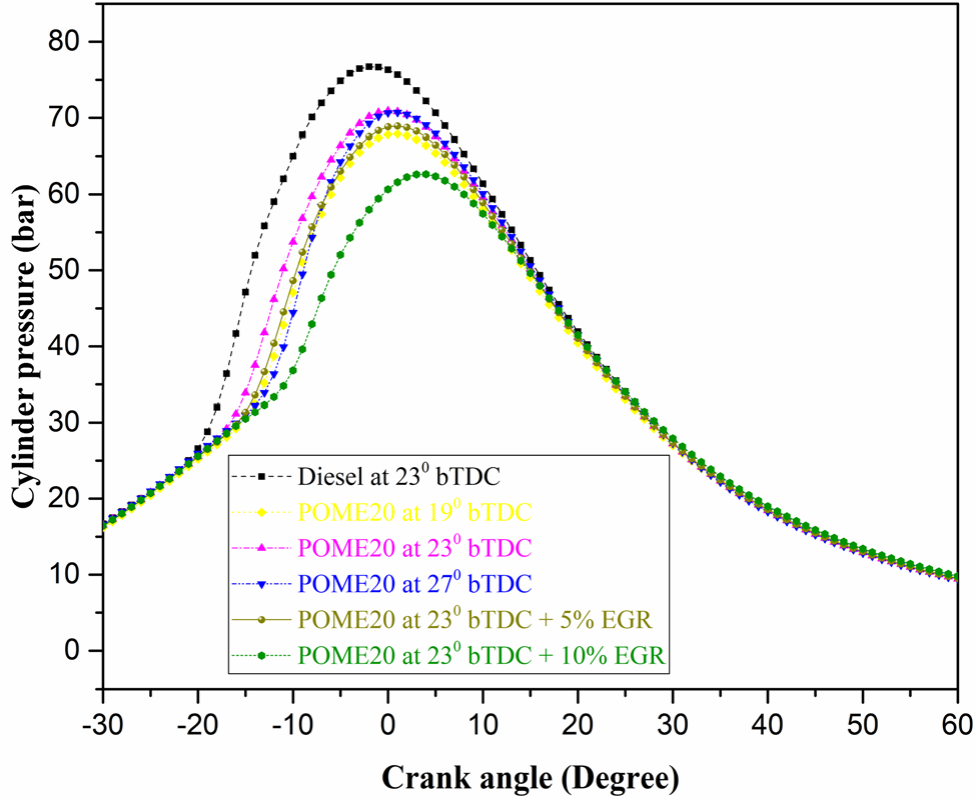
In-cylinder pressure variation with crank angle.

The variation of cylinder pressure for the tested fuels at different ITs and EGR with engine crank angle is delineated in Fig. 11. Cylinder pressure in a diesel engine running on palmyra biodiesel is influenced by injection timing (IT), exhaust gas recirculation (EGR), and load. Advancing IT increases cylinder pressure by allowing more time for combustion before the piston reaches top dead center (TDC), while delaying IT reduces pressure due to late combustion. Higher EGR rates lower cylinder pressure by reducing oxygen availability and slowing combustion, while low EGR has a minimal effect. The pressure increases with the increased load because there is more fuel that is burned. As shown by the combustion data, the pressure in the cylinder at full load is not the same with different types of fuels and operating conditions. Diesel has the highest pressure of 75.2 bar, whereas POME20 has lower pressure depending on the injection timing and EGR use. At 19°bTDC, POME20 reaches 68.2 bar, increasing to 71 bar at 23°bTDC but slightly decreasing to 70.12 bar at 27°bTDC. Introducing EGR at 23°bTDC results in further reductions, with 5% EGR yielding 68.91 bar and 10% EGR dropping to 64.56 bar. Overall, biodiesel blends show lower cylinder pressure than diesel under various injection timings and EGR rates at full load. Moreover, Vellaiyan *et al.*^32^ demonstrated typical RSM best practice that ensued; this correspondence helps to confirm the belief that the enhancements and trade-offs we report are not model artifacts but indicators of actual combustion physics with various biodiesel feedstocks and additive classes.

#### 4.1.4 Heat release rate (HRR)

**Fig. 12:**
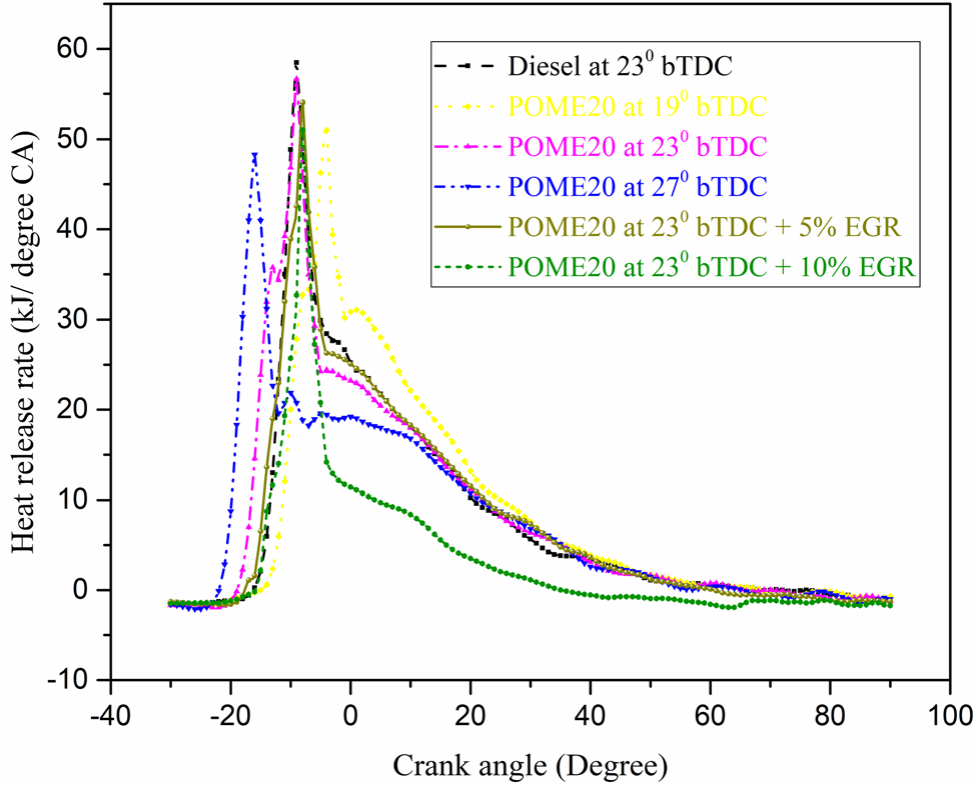
HRR variation with engine crank angles.

The changes in HRR with engine crank angle for the palmyra biodiesel blend is shown in Fig. 12.

The heat release rate (HRR) in a diesel engine running on palmyra biodiesel is influenced by injection timing (IT), exhaust gas recirculation (EGR), and load. The development of IT enhances HRR because combustion begins earlier resulting in quicker energy release towards top dead center (TDC). Latent injection also causes retarded combustion and reduced HRR as a result of late burning. Music Higher EGR decreases HRR through a decrease of oxygen availability and combustion temperature whereas low EGR causes inconsequential effects. The findings indicate that diesel is the fuel that releases the greatest amount of heat per unit of time (HRR). At full load, the HRR values are 61.5 kJ per °CA for diesel, while POME20 at different injection timings shows varying values: 55.98 kJ per °CA at 19°bTDC, 58.86 kJ per °CA at 23°bTDC, and 56.8 kJ per °CA at 27°bTDC. Introducing EGR at 23°bTDC further reduces the HRR, with 5% EGR resulting in 54 kJ per °CA and 10% EGR lowering it to 51 kJ per °CA. The use of EGR at 5% and 10% with POME20 at 23°bTDC leads to a noticeable drop in HRR at full load, indicating reduced combustion intensity due to lower oxygen availability.

#### 4.1.5 Carbon monoxide emissions

**Fig. 13:**
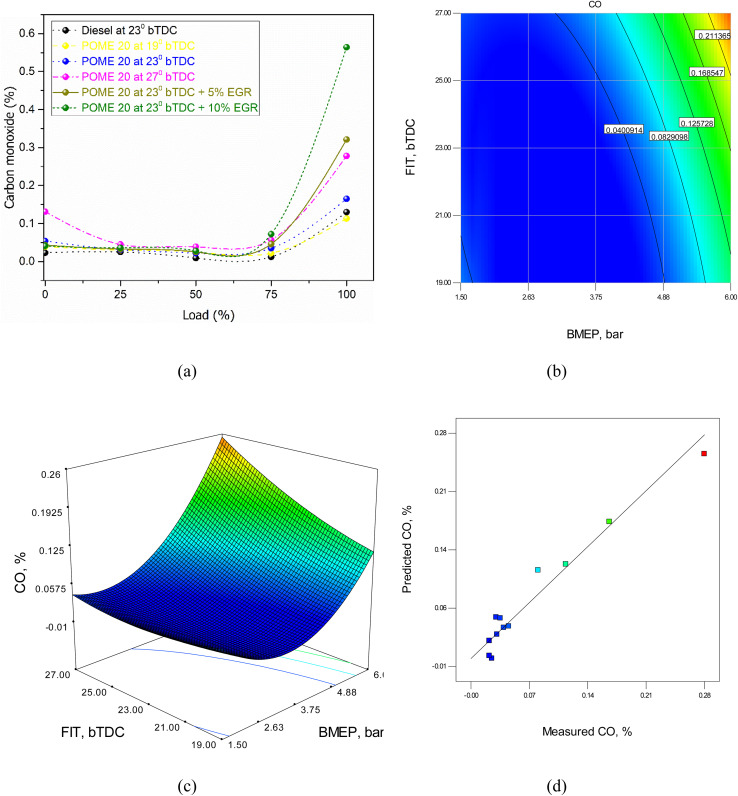
CO model (a) test results (b) 2D contour plot (c) 3D surface plot (d) predicted *vs.* actual values.

It is mainly generated due to incomplete combustion of fuel in the engine, primarily caused by insufficient oxygen, low combustion temperature, poor air-fuel mixing, and inadequate time for combustion. When oxygen is limited, such as with high EGR rates or a rich air-fuel mixture, carbon cannot fully oxidize into carbon dioxide (CO_2_), resulting in CO formation. Low combustion temperatures, often due to delayed injection timing or high EGR, slow down the oxidation process, further contributing to incomplete combustion. Poor air–fuel mixing creates localized rich zones with excess fuel and insufficient oxygen, leading to CO generation. Fig. 13 shows the variation in CO emissions for tested fuels at different injection timings and EGR levels with load. At full load, CO emissions are 0.15% for diesel, while POME20 records 0.132% at 19°bTDC, 0.129% at 23°bTDC, and 0.148% at 27°bTDC. Introducing 5% EGR at 23°bTDC increases CO to 0.195%, and 10% EGR further raises it to 0.216%. CO emissions decrease up to 75% load but rise slightly at higher loads. EGR application results in higher CO emissions compared to diesel and POME20 without EGR, especially at full load.

#### 4.1.6 Unburnt hydrocarbons

**Fig. 14:**
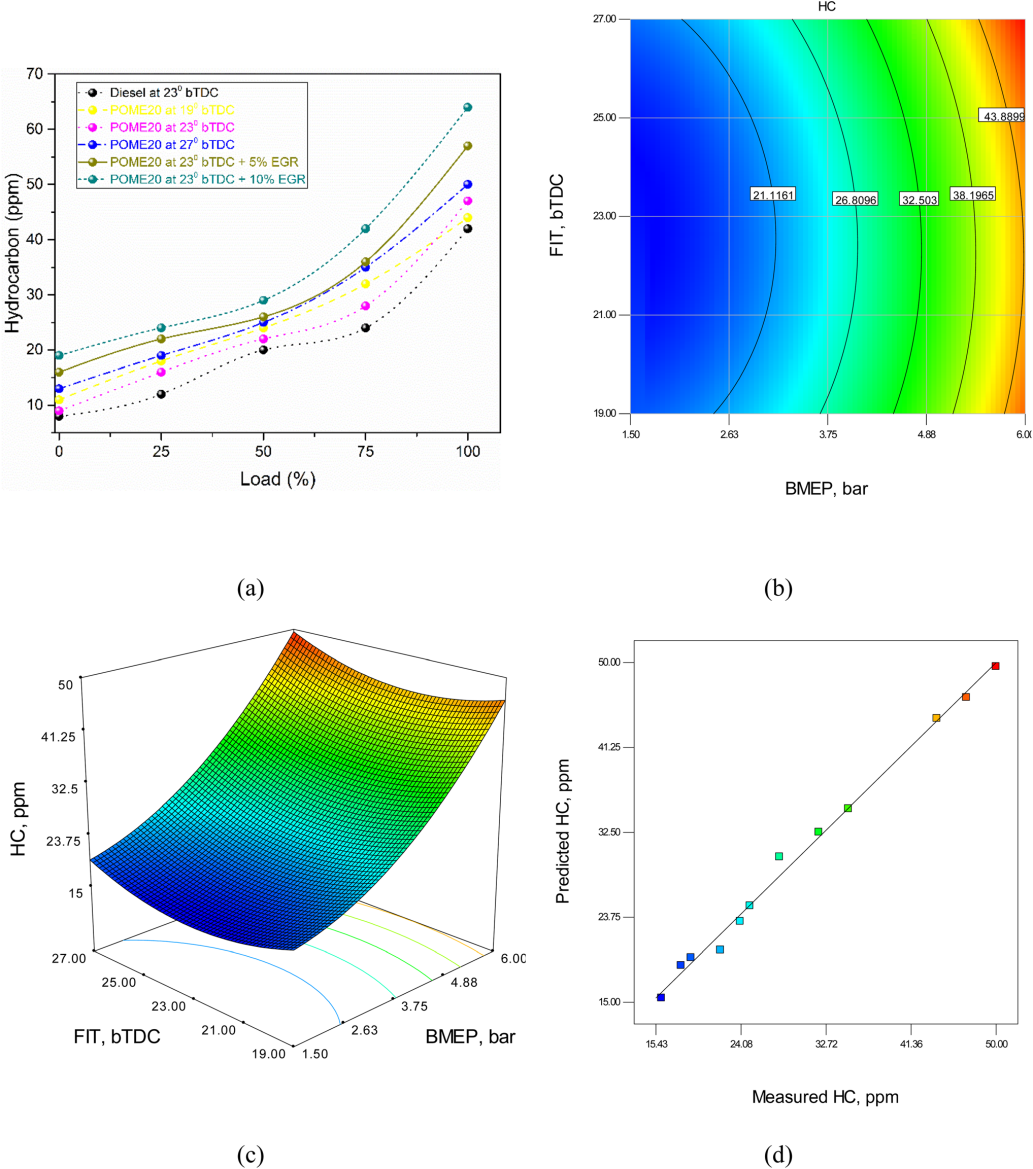
HC model (a) test results (b) 2D contour plot (c) 3D surface plot (d) predicted *vs.* actual values.

Hydrocarbon emissions are generated because of partial combustion process, which results in unburned hydrocarbons escaping into the exhaust. Several factors contribute to this incomplete combustion, such as insufficient oxygen, poor air–fuel mixing, low combustion temperatures, and inadequate time for complete burning. When oxygen is limited often due to high EGR rates or rich air–fuel mixtures the fuel doesn't burn completely, leading to HC formation. Delayed injection timing or rapid cooling of the combustion chamber walls can also leave pockets of unburned fuel, increasing HC emissions. Additionally, at low loads, the lower in-cylinder temperature reduces combustion efficiency, while at high loads, over-fueling can lead to fuel-rich zones, further contributing to HC emissions. Fig. 14 shows the variation in HC emissions with different injection timings and EGR. At full load, hydrocarbon (HC) emissions are 42 ppm for diesel, while POME20 emits 47 ppm at 19°bTDC, 44 ppm at 23°bTDC, and 50 ppm at 27°bTDC. With 5% EGR, emissions rise to 57 ppm, and with 10% EGR to 64 ppm. Diesel and POME20 at 23°bTDC show lower HC emissions, while retarded injection and higher EGR increase emissions, aligning with Ramakrishna Shareef *et al.*^25^

The RSM equation for HC is given byHC = 139.15 − 2.9 × BMEP − 10.78 × FIT + 0.067 × BMEP × FIT + 1.04 × BMEP^2^ + 0.24 × FIT^2^

#### 4.1.7 Nitrogen oxides

Nitrogen oxides (NO_*x*_) emissions in a diesel engine are formed due to the high-temperature combustion process, where nitrogen and oxygen from the air react under extreme heat. When the air–fuel mixture burns at temperatures exceeding 1800 K (about 1527 °C), nitrogen molecules in the air break down and combine with oxygen, forming NO_*x*_. Advanced injection timing increases in-cylinder temperatures, leading to higher NO_*x*_ emissions. Likewise, the high loads lead to more fuel, which produces more heat and enhances the formation of NO_*x*_. Exhaust gas recirculation (EGR) has the capacity to decrease NO_*x*_ by decreasing oxygen levels and peak burn temperature. Nonetheless, it is important to maximize injection timing and EGR to strike a balance between reduction of NO_*x*_ and the overall engine performance.

**Fig. 15:**
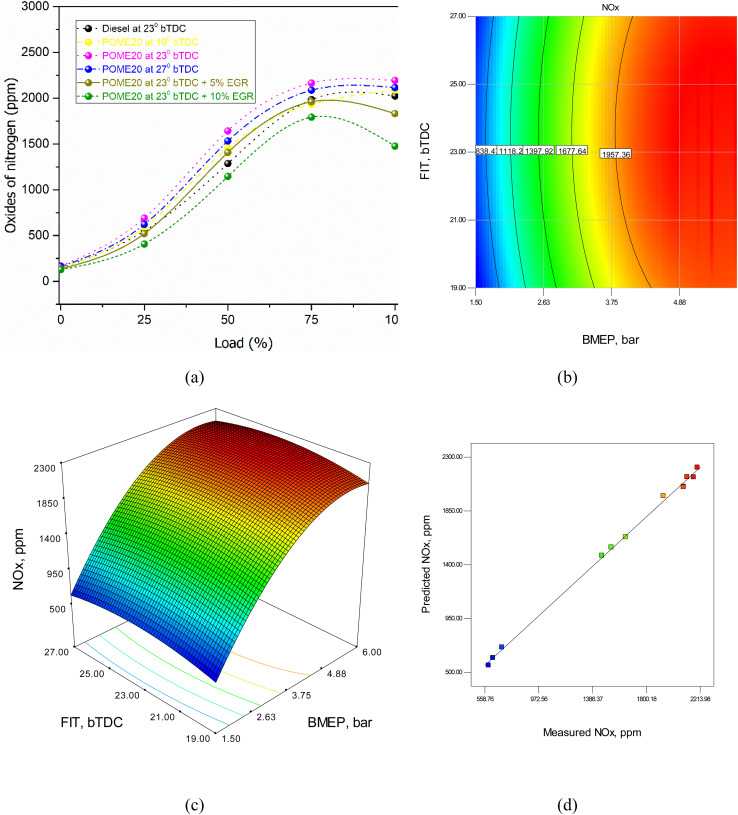
NO_*x*_ model (a) test results (b) 2D contour plot (c) 3D surface plot (d) predicted *vs.* actual values.

Fig. 15 demonstrates the changes in NO_*x*_ emissions at various fuels, injection timing, and EGR concentration. NO_*x*_ emissions are 1624 ppm at full load with POME20 standing at 1829 ppm at 190 bTDC, 1817 at 2300 bTDC and 1843 at 2700 bTDC. The implementation of EGR at 23o bTDc has improved NO_*x*_ to 1431 ppm (5% EGR) and 1275 ppm (10% EGR), which is 11.88% and 21.49% lower than diesel. The results affirm that the progress of injection timing elevates the NO_*x*_ emissions caused by high burner temperatures. Although EGR is effective in lowering NO_*x*_, which decreases oxygen content and high combustion temperature. Shareef *et al.*^29^ and Dhana Raju^40^ have also reported similar trends of NO_*x*_ reduction with EGR, which confirms its usefulness in reducing NO_*x*_ emissions. The RSM equation for BSFC is given byNO_*x*_ = −4765.8 + 1023 × BMEP + 352.43 × FIT + 0.51 × BMEP × FIT − 93.4 × BMEP^2^ − 7.5 × FIT^2^

#### 4.1.8 Smoke opacity

The causes of smoke emissions are poor air–fuel mixture, lack of oxygen, slow combustion, rapid rates of fuel injection, and low combustion temperatures. Smoke emission can be minimized through effective optimization of injection timing, EGR, and air–fuel ratios. Smoke gasses emitted during diesel engines are mainly created as a result of incomplete combustion of the fuel. This occurs when insufficient oxygen does not allow complete oxidation of fuel, which can be caused by high levels of EGR or rich air–fuel ratios. The lack of uniform mixing of air–fuel results in local rich areas, which lead to incomplete combustion and smoke formation. In addition, late injection timing reduces the duration of the combustion process, thus making the fuel incomplete combustion, and high load condition injects more fuel without a commensurate rise in air, resulting in fuel-rich mixtures and smoke. The use of low combustion temperatures also slows down the oxidation rate, which does not burn off all the hydrocarbons. It represents the level of exhausts that is emitted during the exhaust stroke and it is expressed as a percentage.

**Fig. 16:**
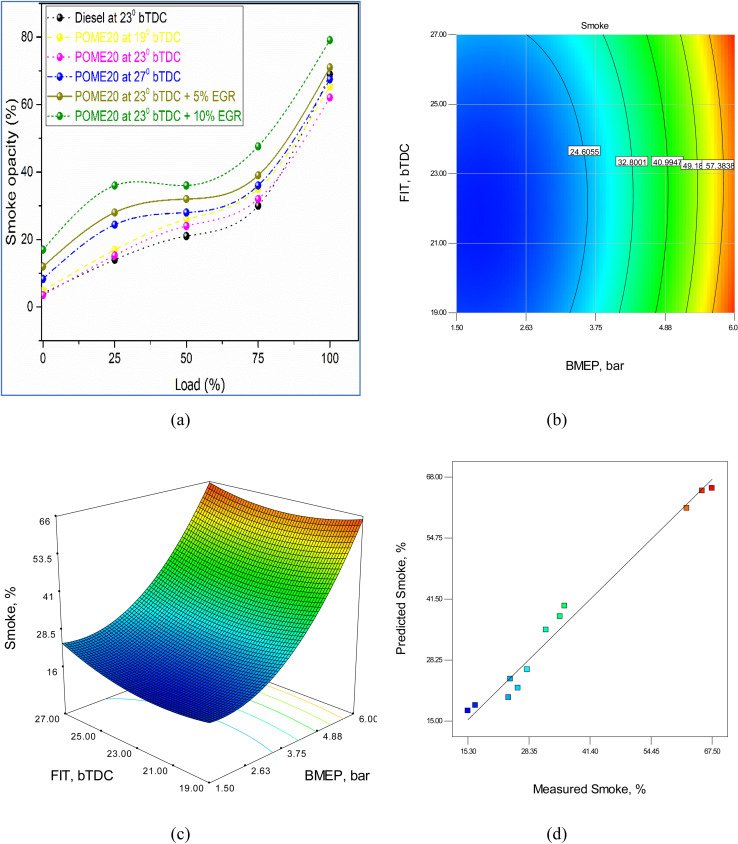
BTE model (a) test results (b) 2D contour plot (c) 3D surface plot (d) predicted *vs.* actual values.

Fig. 16 illustrates the variation in smoke opacity for different fuels, injection timings, and EGR levels across varying loads. At full load, smoke opacity is measured at 75% for diesel, while POME20 shows 67% at 19°bTDC, 69% at 23°bTDC, and 71% at 27°bTDC. Notably, incorporating 5% EGR at 23°bTDC increases opacity to 76%, and 10% EGR pushes it further to 82%. These results highlight that while advancing injection timing slightly reduces smoke opacity due to improved air–fuel mixing, higher EGR rates elevate opacity by limiting oxygen availability, leading to incomplete combustion and greater soot formation.

#### 4.1.9 Desirability based optimization

**Fig. 17:**
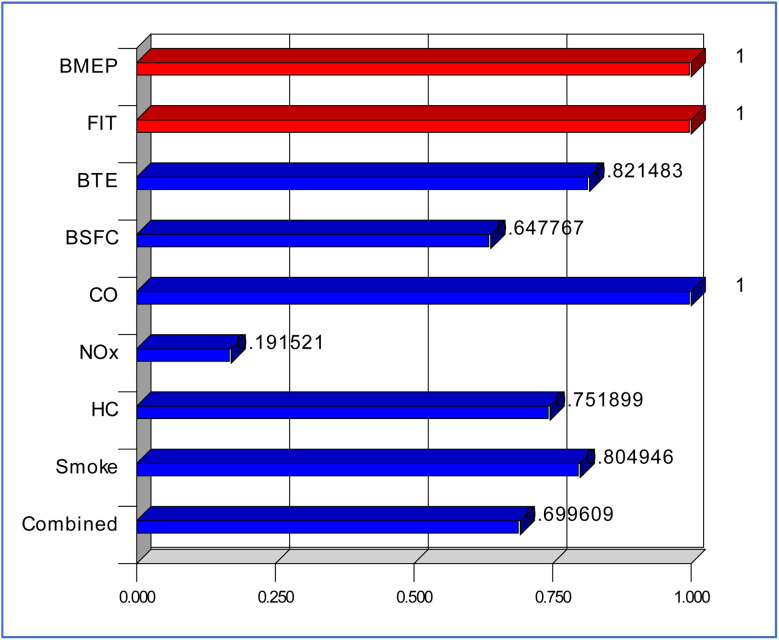
Desirability bar plots.

By use of numerous targets, the desirability-based optimization of the engine data concentrates on obtaining ideal performance and emissions characteristics. The optimization guarantees feasibility under the operating conditions by considering BMEP and FIT inside given ranges (Table 4). Maximizing Brake Thermal Efficiency (BTE) while decreasing Brake Specific Fuel Consumption (BSFC), carbon monoxide (CO), nitrogen oxides (NO_*x*_), hydrocarbons (HC), and smoke emissions is the optimizing aim (Fig. 17). Within their designated ranges, the improved data show BMEP is 3.7 bar and FIT is 21.45°CA. With a high value of 30.05%, BTE shows enhanced energy efficiency close to its highest achievable limit of 32.65%. Better fuel economy is indicated by the minimum BSFC of 0.331 kg kW^−1^ h^−1^. CO is minimized to 0.0064%, NO_*x*_ to 1885.42 ppm, HC to 24.44 ppm, and smoke to 25.482%. Emissions are much lowered. These principles guarantee compliance with environmental criteria since they fit the lower limits of the corresponding emissions bands.

**Table 4:** Optimization results

Name	Goal	Lower limit	Upper limit	
BMEP	Is in range	1.5	6	3.7
FIT	Is in range	19	31	21.45
BTE	Maximize	18.08	32.65	30.05
BSFC	Minimize	0.25	0.48	0.331
CO	Minimize	0.022	0.278	0.0064
NO_*x*_	Minimize	587	2193	1885.42
HC	Minimize	16	50	24.44
Smoke	Minimize	15.3	67.5	25.482
Desirability				0.699

With a 0.699 overall desirability score, the conflicting goals are satisfactorily compromised. Although the result shows certain trade-offs, efficiency and pollution reductions take front stage at the optimal conditions. This optimization system guarantees ecologically friendly and reasonable engine performance.

### 4.2 Statistical results

A statistical method for determining if the means of three or more groups significantly differ is analysis of variance (ANOVA). It separates the observed total variation in the data into elements related to several causes, including random error and treatment effects. In ANOVA, the *F*-test measures statistical significance by contrasting the variance between groups to the variance inside groups. Should the *p*-value be less than a designated significance level—say, 0.05—at least one group mean will clearly vary. In experimental design, model validation, and hypothesis testing ANOVA finds extensive application.

The ANOVA findings offer understanding of the relevance of the model and its elements on Brake Thermal Efficiency (BTE), Brake Specific Fuel Consumption (BSFC), and Carbon Monoxide (CO) emissions (Table 5). With *p*-values 0.0001, the *F*-values for the whole model are rather high for BTE (811.4487) and BSFC (65.54). With an *F*-value of 24.1 and a *p*-value of 0.0007 the CO emissions model also demonstrates relevance. This proves the model's dependability for response prediction. Regarding the factor “A-BMEP,” its effect on every answer is rather very substantial. With *p*-values 0.0001 for the first two and 0.0001 for CO, its *F*-values for BTE, BSFC, and CO correspondingly are 2526.9, 229.25, and 72.2. These data show BMEP as the most important determinant of performance and emissions.

**Table 5:** ANOVA Results for BTE, BSFC, CO

Source	BTE, %			BSFC, kg kW^−1^ h^−1^			CO, %			
	Squares total	Value “*F*”	*p*-value (Prob > *F*)	Squares total	Value “*F*”	*p*-value (Prob > *F*)	Squares total	Value “*F*”	*p*-value (Prob > *F*)	
Model	344.46	811.4487	<0.0001	0.07	65.54	<0.0001	0.06	24.1	0.0007	Significant
A-BMEP	214.54	2526.9	<0.0001	0.048	229.25	<0.0001	0.038	72.2	0.0001	
B-FIT	4.32	50.9	0.0004	0.0012	5.85	0.0519	0.0044	8.47	0.0269	
AB	0.046	0.55	0.4884	0	0	1	0.0061	11.5	0.0144	
*A* ^2^	40.48	476.8	<0.0001	0.0014	6.59	0.0425	0.01606	30.4	0.0015	
*B* ^2^	3.76	44.3	0.0006	0.0008	3.82	0.0983	0.0006	1.2	0.3053	
Residual	0.51			0.0013			0.00316			
Cor total	344.9694			0.071292			0.066634			

The factor “B-FIT” exhibits uneven relevance. Regarding BTE, the *F*-value of 50.9 and *p*-value of 0.0004 point to great relevance. With a *p*-value of 0.0519, which denotes borderline significance, its impact on BSFC is somewhat weak, nonetheless. The *F*-value of 8.47 and the *p*-value of 0.0269 for CO confirm their modest influence. Only for CO, with an *F*-value of 11.5 and a *p*-value of 0.0144, is the interaction term (AB) significant. This implies a noteworthy combined influence on CO emissions of BMEP and FIT. With corresponding *p*-values of 0.0001 and 0.0006, the quadratic terms (*A*^2^) and (*B*^2^), are significant for BTE. But whereas (*B*^2^) is not (*p* = 0.0983), (*A*^2^) is significant (*p* = 0.0425) for BSFC. (*A*^2^) remains significant (*p* = 0.0015) for CO; (*B*^2^) does not. Minimal residuals imply a good match between the model and the data. The study clarifies that BMEP significantly influences all responses whereas FIT and interaction terms have different effects.

The impacts of Brake Mean Effective Pressure (BMEP) and Fuel Injection Timing (FIT) on NO_*x*_ emissions, hydrocarbon (HC) emissions, and smoke opacity are examined by the ANOVA results shown in Table 5. The model is clearly important since the chosen elements clearly affect the outputs since the *p*-values for every answer are less than 0.0001. Furthermore, verifying the strong link between the elements and responses are the *F*-values for the model—654.15 for NO_*x*_, 195.69 for HC, and 59.69 for smoke. BMEP exhibits the most contribution for NO_*x*_ emissions with an *F*-value of 2074.56 and a *p*-value of 0.0001, therefore stressing its major impact. Though less dramatically, FIT also affects NO_*x*_ emissions using an *F*-value of 10.68 and a *p*-value of 0.0171. Not statistically significant are the interaction term (AB) and higher-order effects of FIT (B2) *p* > 0.05. With an *F*-value of 398.72 and *p* < 0.0001, the quadratic term of BMEP (A^2^) significantly influences NO_*x*_ emissions, though.

BMEP once more plays a significant part for HC emissions; its *F*-value is 685.77 and *p* < 0.0001. FIT has a *p*-value of 0.0016, hence it greatly influences HC; their interaction (AB) is still negligible. For BMEP as well as FIT (*A*^2^ and *B*^2^), quadratic terms much help to lower HC. BMEP rules under smoke opacity with an *F*-value of 177.75 and *p* < 0.0001. FIT has a minor impact—*p* = 0.065. While the quadratic term of FIT (*B*^2^) remains negligible, the quadratic term for BMEP (*A*^2^) greatly influences smoke. BMEP is the main determinant factor overall across all the replies. FIT has a secondary effect especially with regard to HC emissions. Interaction terms and other quadratic effects exhibit little relevance, hence stressing BMEP's main linear contributions (Table 6).

**Table 6:** ANOVA results for NO_*x*_, HC, smoke

Source	NO_*x*_, ppm			HC, ppm			Smoke, %			
	Squares total	Value “*F*”	*p*-value (Prob > *F*)	Squares total	Value “*F*”	*p*-value (Prob > *F*)	Squares total	Value “*F*”	*p*-value (Prob > *F*)	
Model	4 347 933.59	654.15	<0.0001	1494.83	195.69	<0.0001	3715.95	59.69	<0.0001	Significant
A-BMEP	2 757 812.29	2074.56	<0.0001	1047.71	685.77	<0.0001	2213.16	177.75	<0.0001	
B-FIT	14 196.13	10.68	0.0171	45.13	29.54	0.0016	63.28	5.08	0.065	
AB	93.03	0.07	0.8002	1.60	1.05	0.3456	7.40	0.59	0.4701	
*A* ^2^	530 040.33	398.72	<0.0001	65.33	42.76	0.0006	415.36	33.36	0.0012	
*B* ^2^	38 480.04	28.95	0.0017	37.50	24.55	0.0026	43.74	3.51	0.11	
Residual	7976.08			9.17			74.71			
Cor total	4 355 909.67			1504.00			3790.65			

### 4.3 ML results

#### 4.3.1 Data correlation analysis

**Fig. 18:**
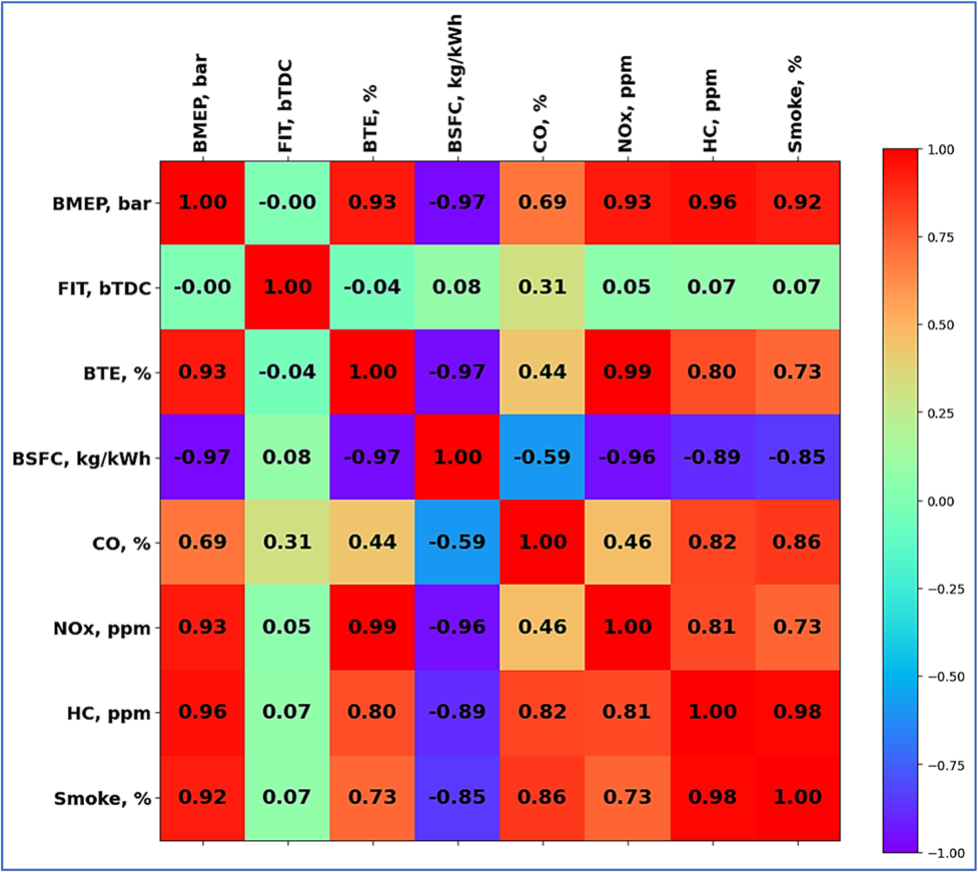
Correlation heat map.

**Fig. 19:**
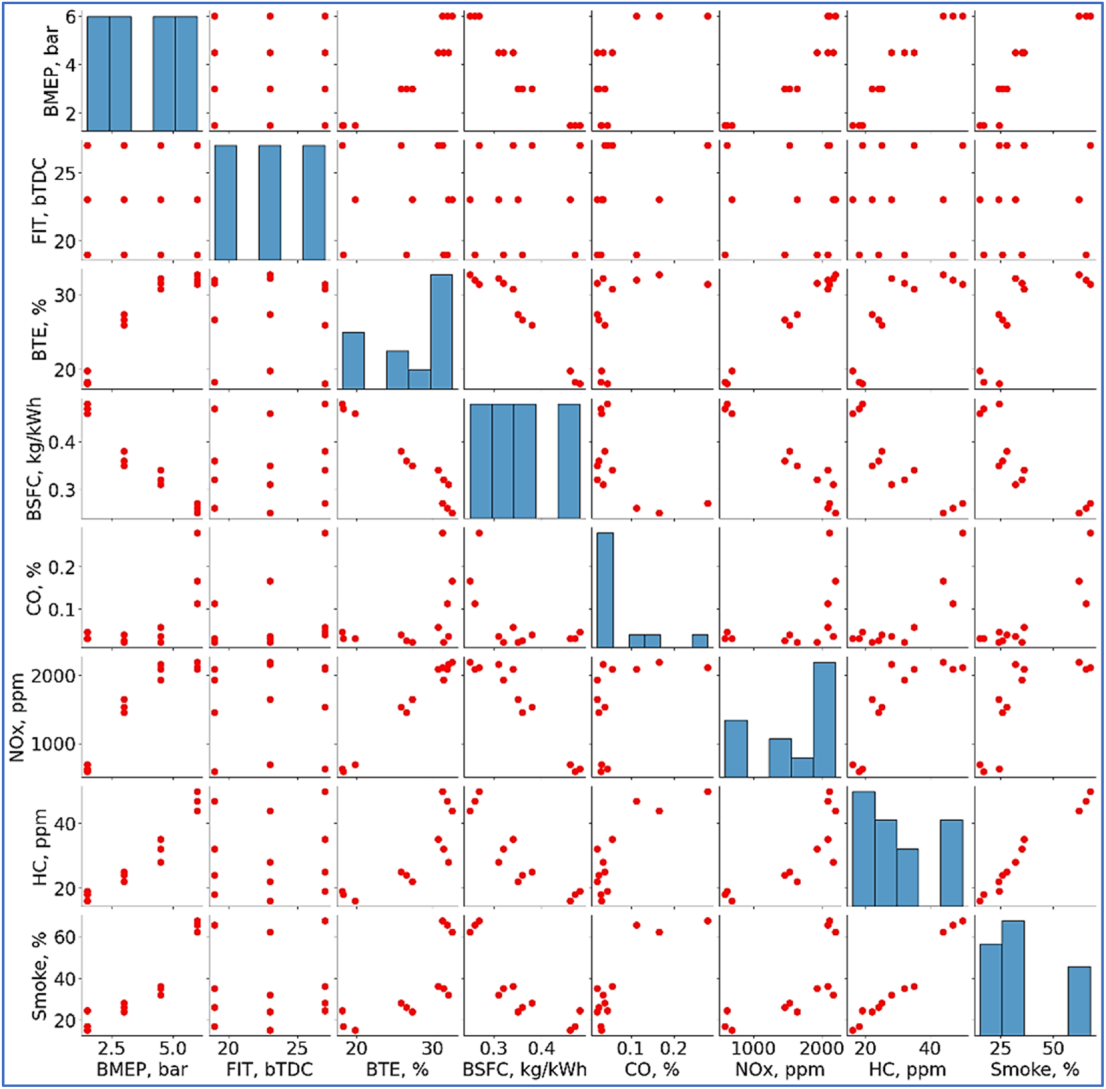
Pairwise correlation plot.

Two visualizations a pair plot and a correlation heat map are shown here (Fig. 18 and 19). Both studies look at interactions among several engine performance and emissions factors. In correlation, with values ranging from −1 (strong negative correlation) to +1 (strong positive correlation), Heat map quantifies linear correlations between parameters. For instance, consistent with increased fuel efficiency at higher pressures, Brake Mean Effective Pressure (BMEP) displays a strong positive association with Brake Thermal Efficiency (BTE, 0.93) and a substantial negative correlation with Brake Specific Fuel Consumption (BSFC, −0.97). Inversely linked to BTE (−0.97, BSFC) reflects that greater efficiency lowers fuel usage. Positive correlations between emission parameters including CO, NO_*x*_, HC, and smoke point to probably interdependence related to combustion properties. Between pair of parameters, the scatter plots expose distributions and possible nonlinear patterns. BTE against BSFC, for example, clearly shows a reverse trend. Emissions measurements (*e.g.*, NO_*x*_*vs.* CO) show scattered clusters implying fluctuation affected by load or fuel type. By aggregating parameter distributions, diagonal histograms emphasize important trends—such as BTE clustering around higher values. These studies taken together provide understanding of performance-emission trade-offs, therefore helping engine development for emissions compliance and efficiency.

#### 4.3.2 Model development

Random Forest and XGBoost were used in predictive modelling with the help of Python libraries with the guarantee of robust and accurate predictions. The XGBoost algorithm has been implemented using the highly efficient XGboost package to deal with complex data structures and large volumes of data. XGBoost employs gradient boosting algorithms to reduce errors successively, therefore, enhancing the performance of the model. Random Forest was implemented using the class sklearn.ensemble. Random Forest Regressor of the sklearn.ensemble sub-domain of the scikit-learn library. This ensemble method of learning splits many decision trees and combines their results, increasing model stability and reducing overfitting. A 5-fold cross-valuation method is used to ensure that the model is evaluated comprehensively in model making. Using the method of KFold provided in the package of sklearn.model, the dataset was divided into five equal folds. In each training, four folds were trained; the other fold was employed as validation. This repetitive process ensured the ability of the model to extrapolate to untouched data, reduced overfitting, and bias.

Evaluation metrics such as *R*^2^, RMSE and MAE were calculated in the module sklearn.metrics on each fold. Random Forest and XGBoost and 5-fold cross-valuation provided a solid foundation on predictive modeling. This approach was found to work well in generating consistent and accurate forecasts in the case of difficult datasets.

#### 4.3.3 BTE model

**Fig. 20:**
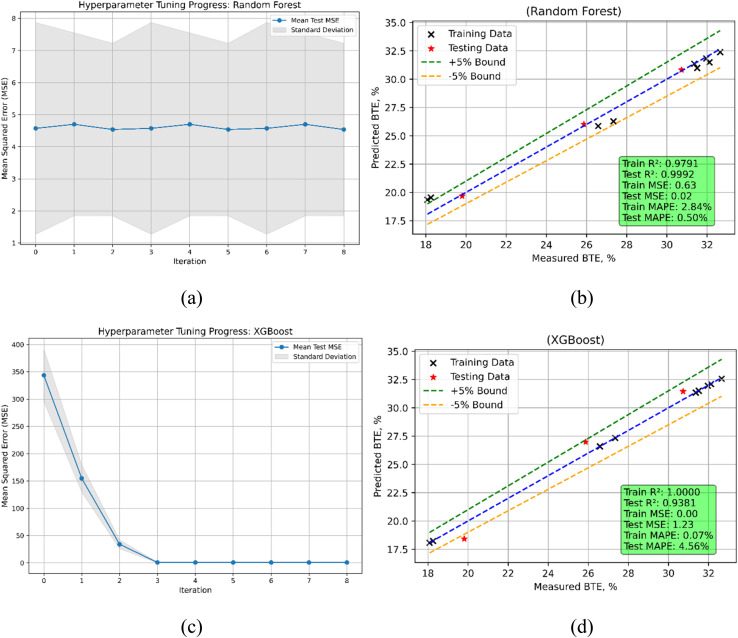
BTE model (a) RF's hyperparameters iterations during training (b) RF's predicted *vs.* actual (c) XGBoost's hyperparameters iterations during training (d) XGBoost's predicted *vs.* actual values.

The performance metrics for Random Forest (RF) and XGBoost models in predicting Brake Thermal Efficiency (BTE) demonstrate notable differences in training and testing phases (Fig. 20). The train MSE for Random Forest is 0.6346; the test MSE is much lower at 0.0152, so indicating great accuracy in both phases (Table 7). For both training and testing datasets, the *R*^2^ scores—which show train: 0.9791, test: 0.9992—indicate that the model explains virtually all the variance in BTE. Particularly during testing, the MAPE values—train: 2.8414%, test: 0.5009%) also demonstrate low prediction errors. Showing great precision on the training set, XGBoost achieves an almost perfect train *R*^2^ (0.99996) and a shockingly low train MSE (0.0011). On the test data, the test MSE is 1.2349 and the test *R*^2^ is 0.9381, so indicating a small decline in predictive performance. Although the training accuracy is rather high, the MAPE values (test: 4.5637%) show that the test predictions show more mistakes than Random Forest even if the training accuracy is quite high. Comparatively to XGBoost for this dataset, Random Forest shows consistent performance across training and testing stages with reduced mistakes and improved generalization to unseen data.

**Table 7:** Statistical measures of the model

Model	ML	Train MSE	Test MSE	Train R2	Test R2	Train MAPE	Test MAPE
BTE	Random forest	0.634559	0.015167	0.979103	0.99924	2.84143	0.500863
	XGBoost	0.001097	1.234873	0.999964	0.938115	0.074854	4.563731
BSFC	Random forest	9.80 × 10^−5^	0.000143	0.984891	0.960447	2.121099	2.821765
	XGBoost	2.47 × 10^−5^	0.000128	0.996195	0.964737	1.169963	2.801107
CO	Random forest	0.000839	0.000423	0.864393	0.883168	13.83041	8.83089
	XGBoost	2.65 × 10^−6^	0.000431	0.999572	0.88083	2.301703	13.39301
NO_*x*_	Random forest	2965.161	8568.507	0.992643	0.898346	3.915176	4.155322
	XGBoost	7.625022	9193.543	0.999981	0.89093	0.100335	3.972084
HC	Random forest	3.165911	19.96987	0.969493	0.894153	4.943921	11.55111
	XGBoost	0.006021	12.95939	0.999942	0.931311	0.155119	6.515858
Smoke	Random forest	7.063656	20.66565	0.977438	0.921586	5.864621	10.63819
	XGBoost	1.05 × 10^−6^	4.589013	1	0.982587	0.002423	3.898767

#### 4.3.4 BSFC model

**Fig. 21:**
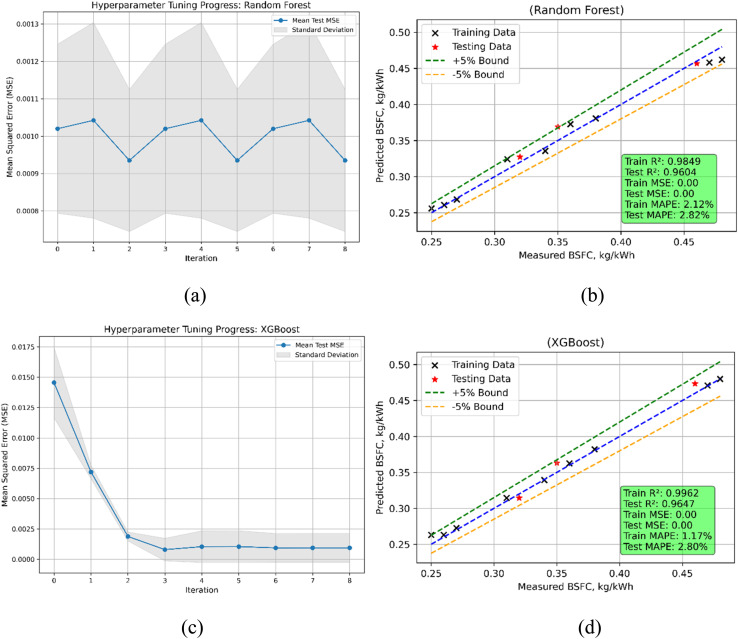
BSFC model (a) RF's hyperparameters iterations during training (b) RF's predicted *vs.* actual (c) XGBoost's hyperparameters iterations during training (d) XGBoost's predicted *vs.* actual values.

The performance metrics for Random Forest (RF) and XGBoost models in predicting Brake Specific Fuel Consumption (BSFC) reveal strong predictive capabilities for both models, with slight differences in accuracy (Fig. 21). For Random Forest, the train MSE is 9.80 × 10^−5^ and the test MSE is slightly higher at 1.43 × 10^−4^. The train *R*^2^ is 0.9849, and the test *R*^2^ is 0.9604, indicating that the model explains over 96% of the variance in BSFC during testing. The MAPE values are 2.12% for training and 2.82% for testing, reflecting low prediction errors. XGBoost achieves a lower train MSE of 2.47 × 10^−5^ and a slightly better test MSE of 1.28 × 10^−4^ compared to Random Forest. The train *R*^2^ of 0.9962 indicates a closer fit to the training data, while the test *R*^2^ of 0.9647 demonstrates marginally better prediction accuracy on unseen data. The MAPE values for XGBoost are 1.17% for training and 2.80% for testing, showcasing its ability to maintain low error rates. Both models perform well, but XGBoost slightly outperforms Random Forest in terms of test MSE, test *R*^2^, and train MAPE, indicating its suitability for precise BSFC predictions in this case.

#### 4.3.5 CO emission model

**Fig. 22:**
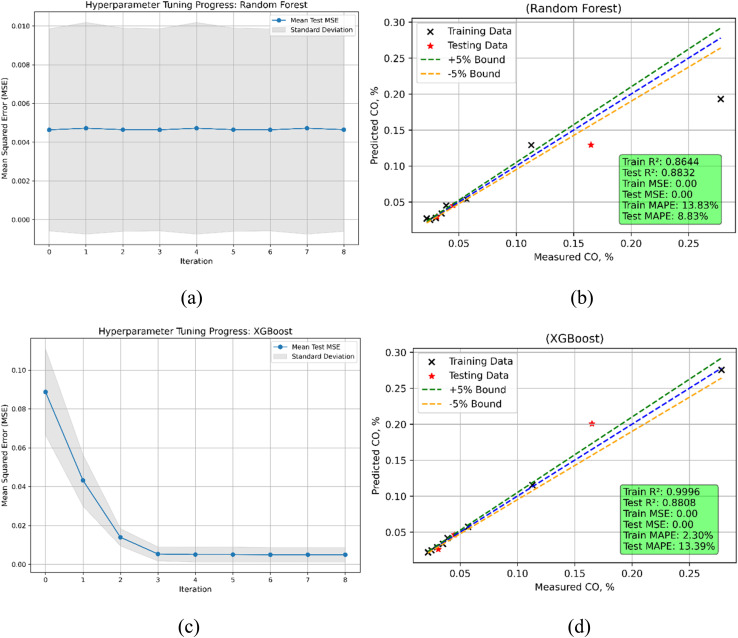
CO emission model (a) RF's hyperparameters iterations during training (b) RF's predicted *vs.* actual (c) XGBoost's hyperparameters iterations during training (d) XGBoost's predicted *vs.* actual values.

Both Random Forest and XGBoost models demonstrate good performance but with quite different results in estimating carbon monoxide (CO) emissions (Fig. 22). Random Forest's train MSE is 0.000839; XGBoost's is substantially lower at 0.000002, suggesting that XGBoost far more precisely fits the training data. On unknown data, the test MSE for both models is rather similar—Random Forest at 0.000424 and XGBoost at 0.000431 suggests comparable generalizing performance. Random Forest shows a modest to strong link between the input variables and the CO emissions in both phases with *R*^2^ value of 0.8644 during training and 0.8832 during testing. Reflecting a modest decline in generalizing ability, XGBoost displays virtually perfect fit in training (*R*^2^ = 0.9996) but a somewhat lower *R*^2^ of 0.8808 on the test data. Relative to training, Random Forest's MAPE is somewhat high—13.83%—but declines to 8.83% during testing, therefore demonstrating significant error reduction on unknown data. At 2.30%, XGBoost demonstrates substantially lower training MAPE; nonetheless, its test MAPE rises dramatically to 13.39%, so emphasizing a larger prediction error in the test phase. XGBoost shines overall in training accuracy; Random Forest shows more consistent performance over testing and training.

#### 4.3.6 NO_*x*_ emission model

**Fig. 23:**
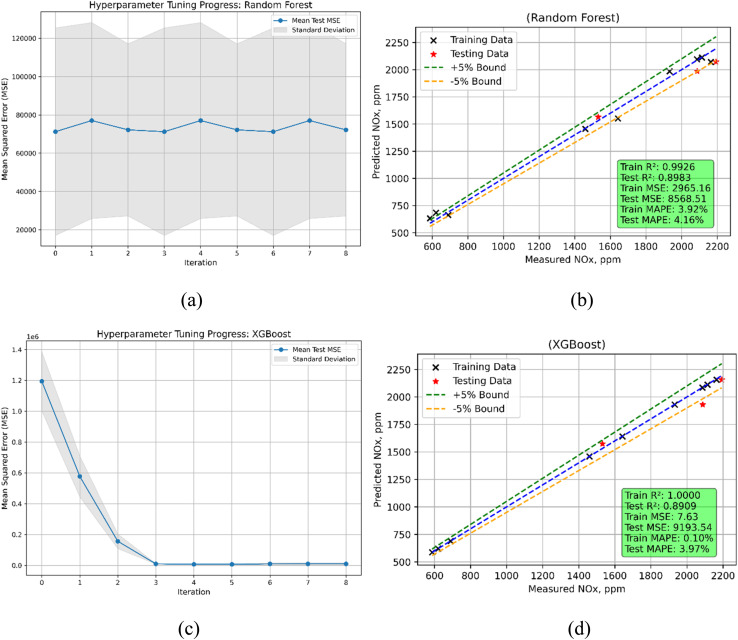
NO_*x*_ emission model (a) RF's hyperparameters iterations during training (b) RF's predicted *vs.* actual (c) XGBoost's hyperparameters iterations during training (d) XGBoost's predicted *vs.* actual values.

Random Forest and XGBoost models show different performance features in nitrogen oxides (NO_*x*_) emission prediction (Fig. 23). While XGBoost obtains a lot closer fit to the training data by means of a substantially lower train MSE of 7.625, the train MSE for Random Forest is 2965.16. Though Random Forest's test MSE of 8568.51 is lower than XGBoost's test MSE of 9193.54, this indicates that Random Forest somewhat better generalizes to unseen data. The *R*^2^ values show how well the models explain variation in NO_*x*_ emissions. With a train *R*^2^ of 0.9926 and a test *R*^2^ of 0.8983 Random Forest shows a great fit during training but a clear decline in predicting accuracy on the test data. Conversely, XGBoost performs well on training data but with a minor decline in test *R*^2^ to 0.8909, implying that it produces nearly perfect *R*^2^ during training (0.99998). Random Forest's MAPE values, in testing and training respectively, are 4.16% and 3.92% respectively, so suggesting low prediction errors. Although XGBoost has a much smaller train MAPE of 0.10%, the test MAPE rises to 3.97%, so stressing a larger inaccuracy when projecting on fresh data. XGBoost performs better on training data overall; Random Forest provides more consistent performance both during training and testing.

#### 4.3.7 HC emission model

**Fig. 24:**
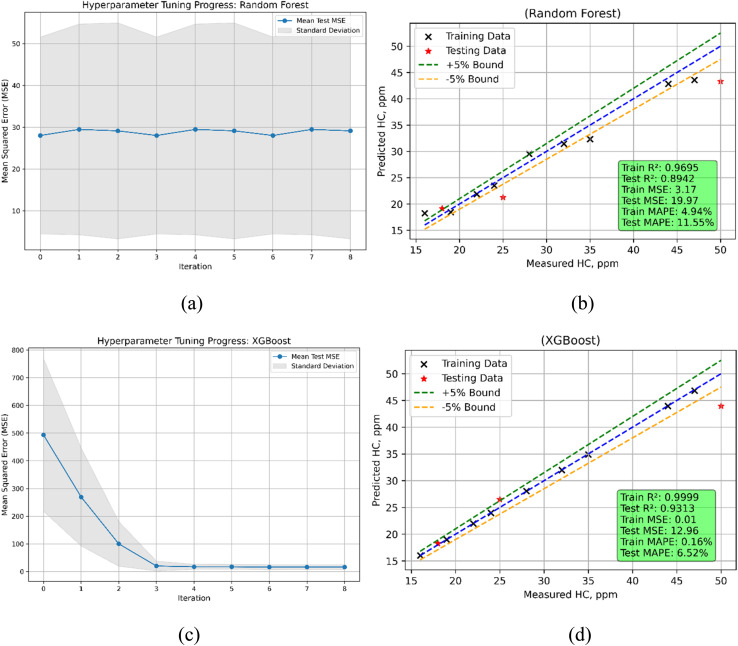
HC emission model (a) RF's hyperparameters iterations during training (b) RF's predicted *vs.* actual (c) XGBoost's hyperparameters iterations during training (d) XGBoost's predicted *vs.* actual values.

In predicting hydrocarbon (HC) emissions, both Random Forest and XGBoost models show considerable differences in performance, with each model excelling in different areas (Fig. 24). Applied to unseen data, the train MSE for Random Forest is 3.1659; the test MSE rises greatly to 19.9699, indicating a notable decline in accuracy. With a far lower train MSE of 0.0060, XGBoost indicates almost ideal fit to the training data. Its test MSE is 12.9594, though, and its generalizing performance falls when compared to Random Forest. Examining the *R*^2^ figures, Random Forest shows high predicting ability in both stages but does poorly on testing with a *R*^2^ of 0.8942 and 0.9695 respectively. Reflecting outstanding training accuracy, XGBoost shows almost flawless train *R*^2^ of 0.9999. Its test *R*^2^ falls to 0.9313, still rather excellent but shows a small decrease in generality. The MAPE values mirror the prediction error percentages. With a quite high train MAPE of 4.94% and a better test MAPE of 11.55%, Random Forest Conversely, XGBoost performs remarkably during training with a very low train MAPE of 0.16%, but the test MAPE rises to 6.52%, suggesting more error during testing than in training. In essence, Random Forest provides more consistent performance across both stages; XGBoost shines in training accuracy.

#### 4.3.8 Smoke emission model

**Fig. 25:**
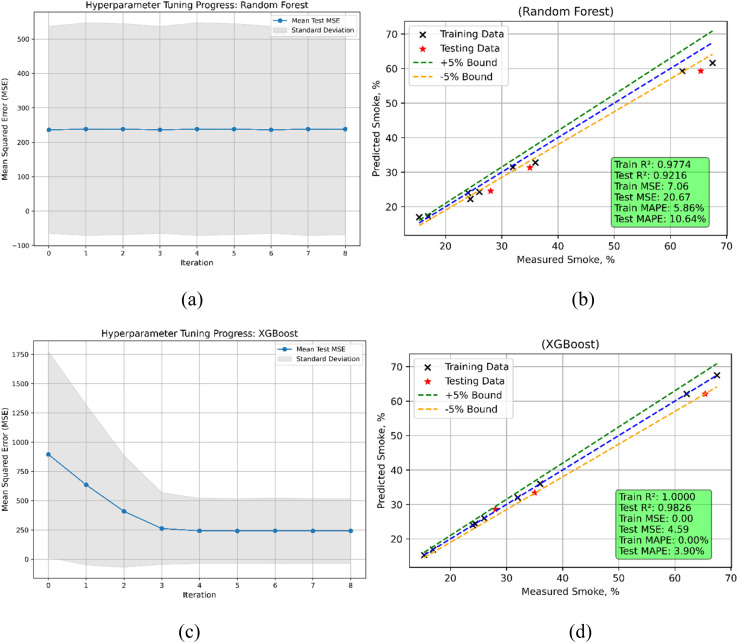
Smoke emission model (a) RF's hyperparameters iterations during training (b) RF's predicted *vs.* actual (c) XGBoost's hyperparameters iterations during training (d) XGBoost's predicted *vs.* actual values.

The results for predicting smoke emissions highlight the differences in performance between the Random Forest (RF) and XGBoost models (Fig. 25). The train MSE for Random Forest is 7.0637; the test MSE rises to 20.6657, thereby demonstrating a decrease in accuracy when forecasting unmet data. Random Forest's *R*^2^ for training and testing is 0.9774 and 0.9216 respectively, indicating that although the model performs somewhat less accurately on the test data, it fits the training data rather well. With a test MAPE of 10.64% and a train MAPE of 5.86%, the relative prediction errors in the test phase seem to be quite greater. Conversely, with a train MSE = 0.000001 and a train *R*^2^ of 1, XGBoost shows almost perfect fit to the training data, hence showing great accuracy. With a test MSE of 4.5890 and a test *R*^2^ of 0.9826, the test indicates a minor decline in performance when compared to the training phase however still shows high generalizing ability. While the test MAPE is 3.90%, which is far lower than Random Forest, the train MAPE is remarkably low at 0.0024%, thereby indicating improved prediction accuracy in the test phase. Regarding MSE, *R*^2^, and MAPE for smoke emissions, XGBoost beats Random Forest both in the training and test phases.

## 5 Conclusion

Experiments on diesel engines fueled with various Palmyra biodiesel blends revealed that POME20 delivered performance closest to diesel. Key findings include:

❖ Among the operated diesel engine conditions, the highest BTE of 32.06% was achieved at 23°bTDC, making it the optimal timing.

❖ Combustion characteristics for POME20 at 23°bTDC showed a HRR of 58.86 J per °CA and a in-cylinder pressure of 71 bar at maximum load, closely resembling diesel performance.

❖ At 23°bTDC, POME20 showed a 14% reduction in CO emissions and an 8% decrease in smoke opacity compared to diesel, though NO_*x*_ emissions were higher with POME blends.

❖ The best performance was observed with POME20 at 23°bTDC and EGR application, reducing NO_*x*_ emissions by 11% and 14% at 5% and 10% EGR rates, respectively, while enhancing BTE.

❖ Among the predictive models, XGBoost slightly outperformed Random Forest in terms of test mean squared error (MSE), test coefficient of determination (*R*^2^), and training mean absolute percentage error (MAPE), demonstrating its superior accuracy for performance and emission prediction.

❖ The ML framework allows real-time optimization for practical use.

❖ POME blends up to 20% can be used in existing diesel engines with minimal modification, enabling partial fossil fuel replacement.

❖ Preliminary results show reduced carbon and particulate emissions, with slight NO_*x*_ rise and upstream impacts requiring further life cycle assessment. Overall, POME demonstrates strong technical and environmental potential as a sustainable CI engine biofuel.

These results highlight POME20's potential as a promising alternative fuel with performance characteristics close to diesel when optimized with proper injection timing and EGR rates.

## Conflicts of interest

The authors declare that they have no conflict of interest.

## Abbreviations

BTE: Brake thermal efficiency
BSFC: Brake specific fuel consumption
CO: Carbon monoxide
HC: Hydrocarbons
NO_*x*_: Nitrogen oxides
SO: Smoke opacity
POME: Palmyra oil methyl ester
POME20: 20% Palmyra oil methyl ester + 80% diesel
ML: Machine learning
RSM: Response surface method
LTC: low temperature combustion
HRR: Heat release rate
RF: Random forest
EGR: Exhaust gas recirculation
bTDC: Before top dead centre
CRDI: Common rail direct injection
CR: Compression ratio
SOI: Start of injection
BMEP: Brake mean effective pressure
IT: Injection timing
RIT: Retarded injection timing
AIT: Advanced injection timing
XGBoost: Extreme gradient boosting
GC: Gas chromatogram
CP: Cylinder pressure
ANOVA: Analysis of variance

## Data Availability

The data that supports the findings of this study are available within the article.
